# Ethephon Reduces Maize Nitrogen Uptake but Improves Nitrogen Utilization in *Zea mays* L.

**DOI:** 10.3389/fpls.2021.762736

**Published:** 2022-01-11

**Authors:** Yushi Zhang, Yubin Wang, Churong Liu, Delian Ye, Danyang Ren, Zhaohu Li, Mingcai Zhang

**Affiliations:** ^1^Engineering Research Center of Plant Growth Regulator, Ministry of Education, College of Agronomy and Biotechnology, China Agricultural University, Beijing, China; ^2^Key Laboratory of Farming System, Ministry of Agriculture of China, College of Agronomy and Biotechnology, China Agricultural University, Beijing, China; ^3^College of Crop Science, Fujian Agriculture and Forestry University, Fujian, China

**Keywords:** ethephon, maize (*Zea mays* L.), nitrogen use efficiency, nitrogen uptake, nitrogen remobilization, plant density

## Abstract

Increasing use of plant density or/and nitrogen (N) application has been introduced to maize production in the past few decades. However, excessive planting density or/and use of fertilizer may cause reduced N use efficiency (NUE) and increased lodging risks. Ethephon application improves maize lodging resistance and has been an essential measure in maize intensive production systems associated with high plant density and N input in China. Limited information is available about the effect of ethephon on maize N use and the response to plant density under different N rates in the field. A three-year field study was conducted with two ethephon applications (0 and 90 g ha^−1^), four N application rates (0, 75, 150, and 225 kg N ha^−1^), and two plant densities (6.75 plants m^−2^ and 7.5 plants m^−2^) to evaluate the effects of ethephon on maize NUE indices (N agronomic efficiency, NAE; N recovery efficiency, NRE; N uptake efficiency, NUpE; N utilization efficiency, NUtE; partial factor productivity of N, PFPN), biomass, N concentration, grain yield and N uptake, and translocation properties. The results suggest that the application of ethephon decreased the grain yield by 1.83–5.74% due to the decrease of grain numbers and grain weight during the three experimental seasons. Meanwhile, lower biomass, ${\text{NO}}_{3}^{-}$ and ${\text{NH}}_{4}^{+}$ fluxes in xylem bleeding sap, and total N uptake were observed under ethephon treatments. These resulted in lower NAE and NUpE under the ethephon treatment at a corresponding N application rate and plant density. The ethephon treatment had no significant effects on the N concentration in grains, and it decreased the N concentration in stover at the harvesting stage, while increasing the plant N concentration at the silking stage. Consequently, post-silking N remobilization was significantly increased by 14.10–32.64% under the ethephon treatment during the experimental periods. Meanwhile, NUtE significantly increased by ethephon.

## Introduction

Maize (*Zea mays* L.) is a staple crop species providing global human food, livestock feed, and industrial raw materials. Increasing plant density and nitrogen (N) application is critical for increasing the global maize yield as part of the “Green Revolution” (Chen et al., 2018). In China, the maize plant density has increased from 1.5 plants m^−2^ in the 1950s to more than 6 plants m^−2^ in 2010 (Li et al., 2016), and the application rate of N fertilizer has increased from 93.3 kg ha^−1^ in the 1970s to 238.2 kg ha^−1^ in the 2000s with an average rate of increase of 50 kg ha^−1^ decade^−1^ (Yang et al., 2013). The North China Plain has a typical intensive agricultural model of winter wheat-summer maize double cropping, contributing almost 30% to the total maize output in China. A stable increase of maize yield in this region can be attributed mainly to high N input and increasing plant density (Li et al., 2016; Chen et al., 2018).

In this context, local Chinese farmers become used to applying more N fertilizer and increasing higher plant densities to increase yields. However, excessive N fertilizer and inordinate plant density increases result in crop lodging, which has been the main obstacle to improving grain yield under conditions of high plant density and N fertilizer (Rajkumara, 2008; Meng et al., 2012; Chen et al., 2018). In addition, high temperature and humid environments during the summer maize growing season often present high lodging risks in the winter wheat-summer maize double-cropping systems. Ethephon (2-chloroethyl phosphonic acid) is a plant growth regulator that promotes desirable qualities in crop plants, including shaping canopy structure and improvements in stem quality (Shekoofa and Emam, 2008; Wiersma et al., 2011; Ye et al., 2016; Zhang et al., 2019b, 2020a), therefore, it is the most widely used plant growth regulator to improve maize resistance to lodging. Ye et al. (2016) suggested that ethephon applications are more efficient for improving maize stalk resistance to bending at higher N fertilizer rates. The ethephon treatment has also been shown to result in significantly greater improvements in the yield at a density of 8.25 plants m^−2^ compared with that under 6.75 plants m^−2^ by reducing the lodging ratio. The beneficial effects of ethephon showed an increasing trend of yield as the plant density increased (Zhang et al., 2017). Currently, ethephon applications have been essential to maize production and occupy the greatest market share (38%) of maize plant growth regulators in China (ICAMA, 2020).

To achieve higher grain yields, improving resource use efficiency, including nitrogen use efficiency (NUE), has become a global sustainable development goal. Although the steady increase in maize grain yield in recent years can be largely attributed to N fertilizer input, excessive N application generally results in low NUE and leads to resource waste and environmental pollution in China (Guo et al., 2010; Ju and Christie, 2011). NUE is referring to the ratio of grain yield to per-unit available N and can be further divided into N uptake efficiency (NUpE) and N utilization efficiency (NUtE) (Xu et al., 2012). The NUpE is limited by the root function and total shoot biomass, whereas NUtE is associated with N translocation to grains, where it contributes to the protein content and thus the grain assimilation capacity, resulting in increased grain yield (Barneix, 2007). Previous studies on the effects of ethephon in maize production have mainly focused on stem lodging resistance and final yield under different N application rates and plant densities (Shekoofa and Emam, 2008; Wiersma et al., 2011; Ye et al., 2016; Zhang et al., 2019a, 2020b). However, the effects of ethephon on maize N use properties have not been well documented.

Ethephon is readily absorbed by the plant and then directly releases ethylene. Ethylene has an important regulatory role in modulating numerous physiological and morphological responses to N availability (Jung et al., 2009; Iqbal et al., 2015). Under laboratory conditions, high nitrate levels induce the production of ethylene, which in turn regulates N uptake by modulating nitrate transporter activities in *Arabidopsis* and maize (Tian et al., 2009; Saiz-Fernández et al., 2017). N deficiency has been shown to promote rapid bursts of ethylene production and activate ethylene signaling to regulate plant nitrate acquisition from the environment (Zheng et al., 2013). Although the connection between ethylene and N use has been revealed at the molecular level under laboratory conditions (Tian et al., 2009; Leblanc et al., 2013; Lemaire et al., 2013; Zheng et al., 2013), little information on this process has been reported under field conditions, including how the use of ethephon may interact with other crop management techniques.

Therefore, in this study, the effects of ethephon application at four rates of N application and two planting densities were assessed under field conditions in three separate years. The objectives of this study were to investigate the effects of ethephon application on N uptake, localization within the plant, and remobilization, and to assess the effects of ethephon on grain yield, grain harvesting index (HI), and relevant N efficiency categories in response to plant density and N application rate. This information contributes to a better understanding of how ethephon regulates maize N use more strategically in combination with optimal N management and plant density practices to improve NUE and therefore maize grain yield under real-field conditions.

## Materials and Methods

### Site Description and Weather Condition

**Figure 1:**
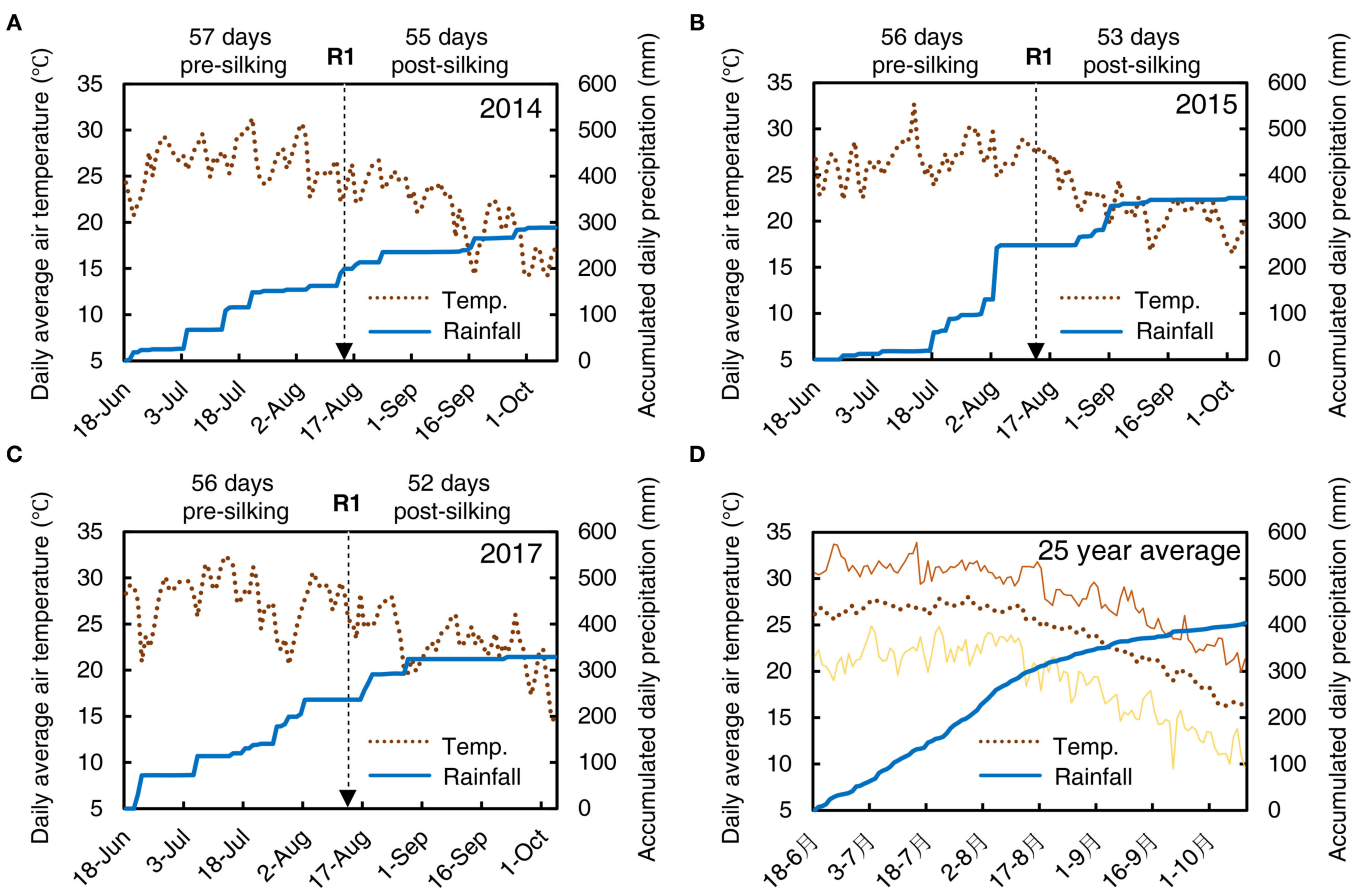
The weather conditions during the summer maize growing season for Wuqiao in 2014 **(A)**, 2015 **(B)**, 2017 **(C)**, and last 25 years average **(D)**. The daily average air temperature (°C, left Y-axis) is shown by a dotted line, and accumulated daily precipitation (mm, right Y-axis) is shown by a wide solid line. The minimum and maximum daily average air temperature in the last 25 years are shown by the lower and upper thin solid lines in **(D)**, respectively. The date for actual 50% silking (R1) in each growing season is marked by a dashed arrow, and days before and after silking are marked at the top of each panel.

All field experiments were carried out at Wuqiao Experimental Station of China Agricultural University (37° 36′ N, 116° 28′ E) located in the North China Plain during 2014, 2015, and 2017 maize growing season. This zone has a typical temperate semi-arid monsoon climate with an annual mean air temperature of 13.1°C and annual mean precipitation of 565 mm. The daily mean air temperature and precipitation during the three maize growing seasons and average in the last 25 years were presented in Figure 1. Similar weather conditions were observed among the three experimental seasons and the 25-years average.

### Experimental Design and Management

A randomized complete block design was employed with six plot replications under each treatment. Each plot was 6 × 6 m in size. The soil type was sandy clay loam (Calcaric Fluvisol), and the chemical characteristics of the 0–30 cm soil profile were tested at the beginning of the experiment on June 11, 2014. The total N content, available phosphorous (P, Olsen-P), available potassium (K), and organic matter content were 0.74 g kg^−1^, 18.8 mg kg^−1^, 127.3 mg kg^−1^, and 12.2 g kg^−1^, respectively. The soil pH was 8.2. Urea was used as an N fertilizer, and the four rates of N application were: none (N0), 75 kg N ha^−1^ (N75), 150 kg N ha^−1^ (N150), and 225 kg N ha^−1^ (N225). In addition, 90 kg P_2_O_5_ ha^−1^ and 90 kg K_2_O ha^−1^ in the form of calcium superphosphate and potassium sulfate were applied to the plots. All fertilizers were applied using a rotary tiller (1GKN-250, Jiangsu, China) before sowing. Fertilization conditions were kept the same in each plot over successive years in this study. To maintain this consistency, the same fertilizer treatments were applied to the plots in the winter wheat growing season. Soil ${\text{NO}}_{3}^{-}$ concentrations before maize sowing in 2014, 2015, and 2017 growing seasons were 6.4, 7.4, and 6.9 mg kg^−1^ in the N0, 8.7, 7.5, and 7.6 mg kg^−1^ in the N75, 10.4, 11.1, and 13.9 mg kg^−1^ in the N150, and 10.8, 12.7 and 14.6 mg kg^−1^ in the N225, respectively.

A widely used local maize variety, Zhengdan958, was used in this study. Seeds were sown manually on June 18, 2014, June 19, 2015, and June 18, 2017. Irrigation of 50 mm was conducted before sowing to promote the emergence of seedlings. The date on which 50% of plants silking was August 14, 2014, August 13, 2015, and August 13, 2017 (marked by a dashed arrow in Figure 1). Ears were harvested on October 8, 2014, October 5, 2015, and October 4, 2017, when the maize had reached physiological maturity. The planting density was 6.75 plants m^−2^ (consistent with the local farmers' plant density used generally, abbreviated to FD) and 7.50 plants m^−2^ (advanced plant density, abbreviated to AD) with the same row spacing of 60 cm. When the maize plants grew to the V8 stage, two rates of ethephon (0 and 90 g ha^−1^ of 200 mg L^−1^ concentration, represented as CT and ET) were applied once through foliar treatments with an automatic sprayer on July 21, 2014, July 22, 2015, and July 19, 2017. The concentrations and treatment time were based on pre-experiment conducted by our group, in which three ethephon concentrations of 200, 400, and 800 mg L^−1^ and seven application times from V7 to V13 were tested (Wang, 2013). The highest yield was observed under the ethephon treatment at V8 with a concentration of 200 mg L^−1^. Other field management practices were applied according to local recommendations.

### Plant Sampling, Biomass, N Content, and Yield Measurements

Above-ground parts of seven typical maize plants were collected from three plots in each treatment at both the silking stage and the harvesting stage. At the harvesting stage, plants were dissected into two parts: grains and stovers. All samples were dried to a constant weight with a drying oven (DHG9248A, Shanghai, China) at 80°C, after which the plant biomass was determined. Pre-silking biomass (PreBM) was defined as the total plant dry matter per unit area at the silking stage, and post-silking biomass (PostBM) was defined as the net difference in plant biomass per unit area between the silking and the harvesting stage. The harvest index (HI) was calculated as the ratio of grain biomass to total plant biomass. The dry samples were ground with an electric grinder (TST-300, Tianjin, China) to give samples from which to determine N concentration according to the Kjeldahl method (Bremner and Mulvaney, 1982), modified for plant samples. Plant N content was calculated as the product of N concentration and corresponding dry weight. The total plant N concentration was calculated as the weighted mean of the N concentration in the grain and stovers.

Maize ears were harvested from the two 5-m-long rows in the middle of the remaining three plots to determine the yield and yield components including ears per hectare and grain number per ear. Grains were also oven-dried at 80°C to constant weight for determination of 1000-grain weight. The grain yield was calculated with 14% moisture content.

### N and N Use Efficiency Assessing Indices Calculation

The sources of plant and grain N allocations were defined and estimated with the following formulas according to Ciampitti and Vyn (2013):

Total N uptake, $NUT\;\left(\text{kg}\;{\text{ha}}^{\text{- 1}}\right)=\;N_{harvest}\;\times \;PD$
Pre-silking N uptake, $PreN\;\left(\text{kg}\;{\text{ha}}^{\text{- 1}}\right)=\;N_{silk}\;\times \;PD$
Post-silking N uptake, ***PostN*** (**kg** **ha**^**- 1**^) **=** ***NUT*** **−** ***PreN***
Post-silking N remobilization, $RemN\;\left(\text{kg}\;{\text{ha}}^{\text{- 1}}\right)=\;{PreN-N}_{stover}\;\times \;PD$
Pre-silking N uptake per plant, ${PreN}_{p}\;\left(\text{g}\;{\text{plant}}^{\text{- 1}}\right)=\;N_{silk}$
Post-silking N uptake per plant, ${PostN}_{p}\;\left(\text{g}\;{\text{plant}}^{\text{- 1}}\right)=\;N_{harvest}-N_{silk}$
Post-silking N remobilization per plant, ${RemN}_{p}\left(\text{g}\;{\text{plant}}^{\text{- 1}}\right)=\;N_{silk}-N_{stover}$
Vegetative N remobilization efficiency, ***PVRE*** (**%**) **= *100*% ×** ***RemN*** **/*****PreN***
N harvest index, ***NHI*** (**%**)** = *100*%** **×** ***N***_***grain***_** / *****N***_***harvest***_

Where PD is the plant density (plant ha^−1^); N_harvest_ and N_silk_ is total plant N content at the harvesting and silking stage respectively (g plant^−1^); N_stover_ and N_grain_ is N content of stover and grains at the harvesting stage respectively (g plant^−1^).

The N use efficiency (NUE) assessing indices used in this paper were defined and calculated as follows, according to the equations employed by previous studies (Ciampitti and Vyn, 2012; Xu et al., 2012; Chen and Vyn, 2017):

N agronomic efficiency, $NAE\;\left(\text{kg}\;{\text{kg}}^{\text{- 1}}\right)=\;{\Delta GY\;/\;N}_{applied}$
N recovery efficiency, ***NRE*** **(%)** = ***100*****%** × **Δ** ***NUT / N_applied_***
N uptake efficiency, $NUpE\;\left(\text{kg}\;{\text{kg}}^{\text{- 1}}\right)=\;{NUT\;/\;N}_{applied}$
N utilization efficiency, ***NUtE*** (**kg** **kg**^**- 1**^) **=** ***GY*****/*****NUT***
Partial factor productivity of N, $PFPN\left(kg\;{kg}^{-1}\right)=\;{GY\;/\;N}_{applied}\;=\;NUpE\;\times \;NUtE$

Where GY is the grain yield, ΔGY is the net increased grain yield of the maize with and without N fertilization applied (kg ha^−1^). ΔNUT is the net increased total plant N uptake with and without N fertilization applied (kg ha^−1^). N_applied_ is the N rate applied (kg ha^−1^).

### Xylem Bleeding Sap Collection and Analysis

The maize xylem bleeding sap was collected according to the modification of the process described by Guan et al. (2014). Five plants were sampled at V13 and 30DAS; each plant was cut at the second elongated internode approximately 10 cm above the soil level at 6:00 pm, and the bleeding sap was accumulated for 12 h. The volume of bleeding sap was determined, and then filtered through a 0.45 μm syringe-driven filter and stored at −20°C for inorganic N concentration analysis. The ${\text{NO}}_{3}^{-}$ and ${\text{NH}}_{4}^{+}$ concentrations in bleeding sap were measured using AA3 Continuous Flow Analytical System (Seal, Germany). The ${\text{NO}}_{3}^{-}$ and ${\text{NH}}_{4}^{+}$ fluxes (mg h^−1^ plant^−1^) were calculated by the following equation:


$$\begin{array}{l} {NO}_{3}^{-}\;\left({NH}_{4}^{+}\right)fluxes\;\left(mg\;h^{-1}{plant}^{-1}\right)=\;\\ {NO}_{3}^{-}\;\left({NH}_{4}^{+}\right)\;concentration\;\times \;bleeding\;sap\;volume/\;\\ collecting\;time. \end{array}$$

### Statistical Analysis

Ethephon treatment, N application rate, and plant density were all considered fixed factors in our analysis. Three-way ANOVA was conducted separately for each growing season using the “PROC GLM” procedure in SAS version 9 (SAS Institute Ltd., USA). Fisher's least significant difference (LSD) test at the 0.05 level of probability was carried out to detect the significance of the differences among means between treatment levels. Data for the different factors except ET and CT were jointly used in correlation and regression analysis by PROC REG (SAS software 9, SAS Institute Ltd., USA), and the slopes and intercepts of regression lines for CT and ET were compared based on F-test (*P* < 0.05). A linear plateau model of fit was chosen to simulate the relationship between grain yield and N application rate using the SAS procedure (Cerrato and Blackmer, 1990; Chen et al., 2015).

## Results

### Plant Biomass and Nitrogen Concentration

Ethephon, N rate, and plant density all substantially influenced plant biomass and plant N concentration at both silking and harvesting stages (Table 1). Generally, the biomass at silking and harvest stage per hectare was higher under AD than that under FD conditions, while the N concentrations in the whole plant and the different organs were lower under AD than FD conditions. At increasing levels of N application, plant biomass at the silking stage and stover biomass at the harvesting stage also significantly increased, while the differences in grain weight between N150 and N225 were not significantly different in either 2014 or 2015. Increasing the N rate significantly increased the whole plant N concentrations at the silking stage and N concentrations in both stover and grain at the harvesting stage in all three growing seasons. Ethephon applications significantly reduced plant biomass per hectare at the silking stage by 8.29, 8.13, and 7.27%, stover biomass per hectare at the harvesting stage by 7.82, 4.79, and 10.30%, and grain weight per hectare by 3.77, 6.10, and 5.22% in 2014, 2015, and 2017, respectively. Interestingly, the ethephon treatments significantly increased N concentration at the silking stage by 8.32, 7.34, and 7.28%, but decreased stover N concentration by 11.58, 8.39, and 5.32, in 2014, 2015, and 2017, respectively. The grain N concentration decreased under the ethephon treatment in all three growing seasons, however, these trends were not significant in any of the years.

**Table 1:** The effects of ethephon, N application rate, and plant density on plant biomass and N concentration of the whole plant, stover, and grain at the silking or harvesting stages in maize in 2014, 2015, and 2017.

**Year**	**Treatment**	**BM**_**silk**_		**BM**_**stover**_		**BM**_**grain**_		**BM**_**harvest**_		**NC**_**silk**_		**NC**_**stover**_		**NC**_**grain**_		**NC**_**harvest**_	
		**(t ha** ^**−1**^ **)**		**(t ha** ^**−1**^ **)**		**(t ha** ^**−1**^ **)**		**(t ha** ^**−1**^ **)**		**(mg g** ^**−1**^ **)**		**(mg g** ^**−1**^ **)**		**(mg g** ^**−1**^ **)**		**(mg g** ^**−1**^ **)**	
2014																	
	PGR																
	CT	7.3	a	8.8	a	10.4	a	19.2	a	14.3	b	7.5	a	10.7	a	9.2	a
	ET	6.7	b	8.1	b	10.0	b	18.1	b	15.5	a	6.6	b	10.9	a	9.0	a
	Nitrogen																
	N0	6.1	d	7.3	d	8.7	c	16.1	d	12.1	d	5.1	d	9.3	d	7.4	d
	N75	6.8	c	8.4	c	10.3	b	18.6	c	14.3	c	6.6	c	10.9	c	9.0	c
	N150	7.4	b	8.9	b	10.8	a	19.7	b	16.0	b	7.8	b	11.3	b	9.7	b
	N225	7.9	a	9.3	a	11.0	a	20.3	a	17.4	a	8.6	a	11.9	a	10.4	a
	Density																
	FD	6.5	b	8.1	b	9.4	b	17.5	b	15.7	a	6.9	b	11.3	a	9.3	a
	AD	7.6	a	8.9	a	10.9	a	19.9	a	14.1	b	7.1	a	10.4	b	8.9	b
	Source of variance (a = 0.05)																
	P	^***^		^***^		^*^		^***^		^***^		^***^		ns		ns	
	N	^***^		^***^		^***^		^***^		^***^		^***^		^***^		^***^	
	D	^***^		^***^		^***^		^***^		^***^		^**^		^***^		^**^	
	P × N	ns		^***^		ns		^**^		^***^		^***^		ns		ns	
	P × D	ns		ns		ns		ns		^***^		^***^		ns		ns	
	N × D	^*^		^***^		ns		^***^		^***^		^***^		^**^		ns	
	P × N × D	ns		^**^		ns		ns		^**^		^***^		ns		ns	
2015																	
	PGR																
	CT	7.5	a	8.1	a	9.7	a	17.8	a	12.7	b	6.4	a	10.9	a	8.8	a
	ET	6.9	b	7.7	b	9.1	b	16.8	b	13.6	a	5.9	b	10.8	a	8.5	b
	Nitrogen																
	N0	6.2	d	6.7	d	7.9	c	14.6	c	11.5	d	4.7	d	9.5	d	7.3	d
	N75	7.0	c	8.1	c	9.5	b	17.6	b	12.5	c	5.5	c	10.7	c	8.3	c
	N150	7.5	b	8.4	b	10.1	a	18.4	a	13.6	b	6.5	b	11.3	b	9.1	b
	N225	7.9	a	8.5	a	10.0	a	18.5	a	15.0	a	7.8	a	11.9	a	10.0	a
	Density																
	FD	6.8	b	8.0	a	9.0	b	17.1	b	14.2	a	6.3	a	11.1	a	8.9	a
	AD	7.5	a	7.8	b	9.7	a	17.5	a	12.1	b	5.9	b	10.6	b	8.5	b
	Source of variance (a = 0.05)																
	P	^***^		^***^		^***^		^***^		^***^		^***^		ns		^***^	
	N	^***^		^***^		^***^		^***^		^***^		^***^		^***^		^***^	
	D	^***^		^***^		^***^		^***^		^***^		^***^		^***^		^***^	
	P × N	^**^		^*^		ns		^*^		^*^		^***^		^***^		^*^	
	P × D	^**^		^*^		^*^		^***^		ns		ns		^***^		ns	
	N × D	ns		ns		^***^		^***^		ns		ns		^***^		^***^	
	P × N × D	ns		ns		ns		ns		ns		ns		ns		ns	
2017																	
	PGR																
	CT	7.8	a	8.7	a	9.8	a	18.5	a	13.8	b	6.4	a	10.9	a	8.8	a
	ET	7.3	b	7.8	b	9.2	b	17.1	b	14.9	a	6.1	b	10.7	a	8.6	a
	Nitrogen																
	N0	4.7	d	5.7	d	6.0	c	11.7	d	11.5	d	4.8	d	8.9	d	6.9	d
	N75	7.7	c	8.2	c	9.5	b	17.7	c	13.5	c	5.8	c	9.7	c	7.9	c
	N150	8.7	b	9.4	b	11.0	a	20.4	b	15.6	b	6.7	b	11.7	b	9.4	b
	N225	9.0	a	9.9	a	11.4	a	21.4	a	16.8	a	7.7	a	13.0	a	10.5	a
	Density																
	FD	7.2	b	8.3	b	9.1	b	17.4	b	15.0	a	6.1	a	11.0	a	8.7	a
	AD	7.9	a	8.2	a	9.9	a	18.2	a	13.7	b	6.3	b	10.7	b	8.7	b
	Source of variance (a = 0.05)																
	P	^***^		^***^		^**^		^***^		^***^		^***^		ns		ns	
	N	^***^		^***^		^***^		^***^		^***^		^***^		^***^		^***^	
	D	^***^		ns		^***^		^**^		^***^		^***^		^***^		^***^	
	P × N	^***^		^*^		^*^		^**^		^*^		^***^		ns		ns	
	P × D	ns		ns		ns		ns		ns		^**^		ns		ns	
	N × D	ns		ns		ns		ns		^***^		^***^		^***^		ns	
	P × N × D	ns		ns		ns		ns		ns		^*^		ns		ns	

*CT and ET represented control and ethephon; FD and AD represented farmers' plant density (6.75 plant m^−2^) and advanced plant density (7.5 plant m^−2^); N0, N75, N150, and N225 indicated N application rates at 0, 75, 150, 225 kg N ha^−1^, respectively. BM_silk_, plant biomass at the silking stage; BM_stover_, stover biomass at the harvesting stage; BM_grain_, grain biomass at the harvesting stage; BM_harvest_, plant biomass at the harvesting stage; NC_silk_, plant N concentration at the silking stage; NC_stover_, N concentration in stover at the harvesting stage; NC_grain_, N concentration in grain at the harvesting stage; NC_harvest_, total plant N concentration at the harvesting stage. Within ethephon, N application rate or plant density, values followed by the same letters in the same volume indicated no significant difference (P < 0.05). And ns indicated not significant (P > 0.05)*,

*, **, *** *indicated significant at P < 0.05, 0.01, and 0.001, respectively*.

**Figure 2:**
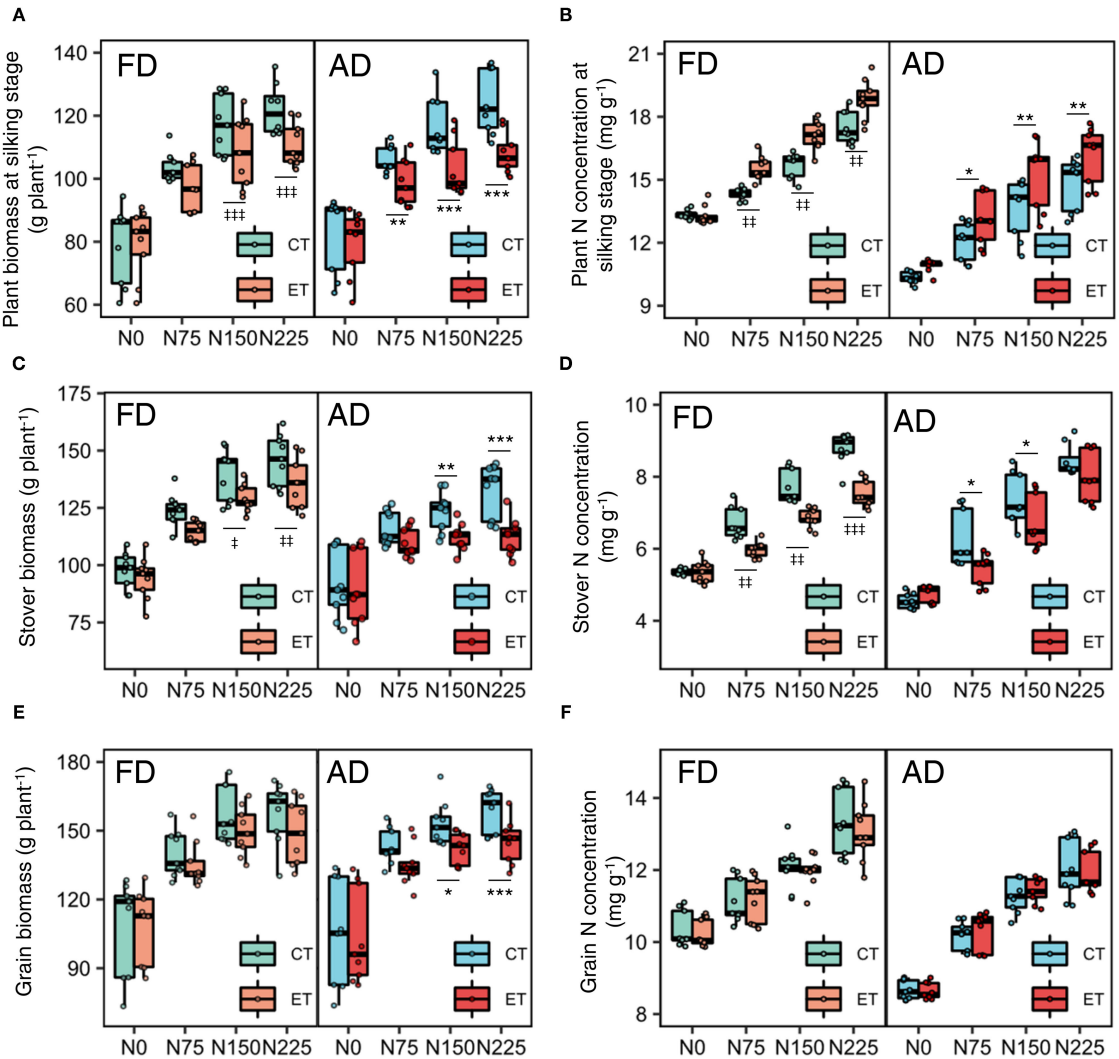
Effects of ethephon on maize biomass and N concentration in the whole plant, stover, and grain under different N application rates and plant densities during 2014-2017 growing seasons. **(A,B)** indicate the plant biomass and nitrogen (N) concentration at silking stage. **(C,D)** indicate the stover biomass and N concentration at the harvesting stage. **(E,F)** indicate the grain biomass and N concentration at the harvesting stage. Data points represent multi-year data under each treatment factor, and the thick lines, lower and upper edges, whiskers outside the boxes represent means, 25th and 75th, 5th and 95th percentiles of all data, respectively. CT and ET represent control and ethephon treatment, respectively; FD and AD represent farmers' plant density (6.75 plant m^−2^) and advanced plant density (7.5 plant m^−2^), respectively. N0, N75, N150, N225 were N application rates at 0, 75, 150, 225 kg N ha^−1^ respectively. *(^‡^), **(^‡‡^), ***(^‡‡‡^) means significant differences between CT and ET under AD(FD) determined by Fisher's least significant difference (LSD, *a* = 0.05) at *P*-value for *F*-test < 0.05, < 0.01 and < 0.001, respectively.

Ethephon and N rate had an interactive effect on biomass and N concentration in each year, but fewer ethephon × plant density interactive effects were observed. Figure 2 shows the value distribution of each parameter during the growing season in each of the three years. Increased N application rates enhanced the negative effects of the ethephon treatment on single plant biomass (Figures 2A–C) and enhanced the positive effects of ethephon on plant N concentration at the silking stage (Figure 2D). The effects of ethephon on plant biomass and N concentration were similar under AD and FD conditions.

### Nitrogen Uptake, Location, and Remobilization

**Figure 3:**
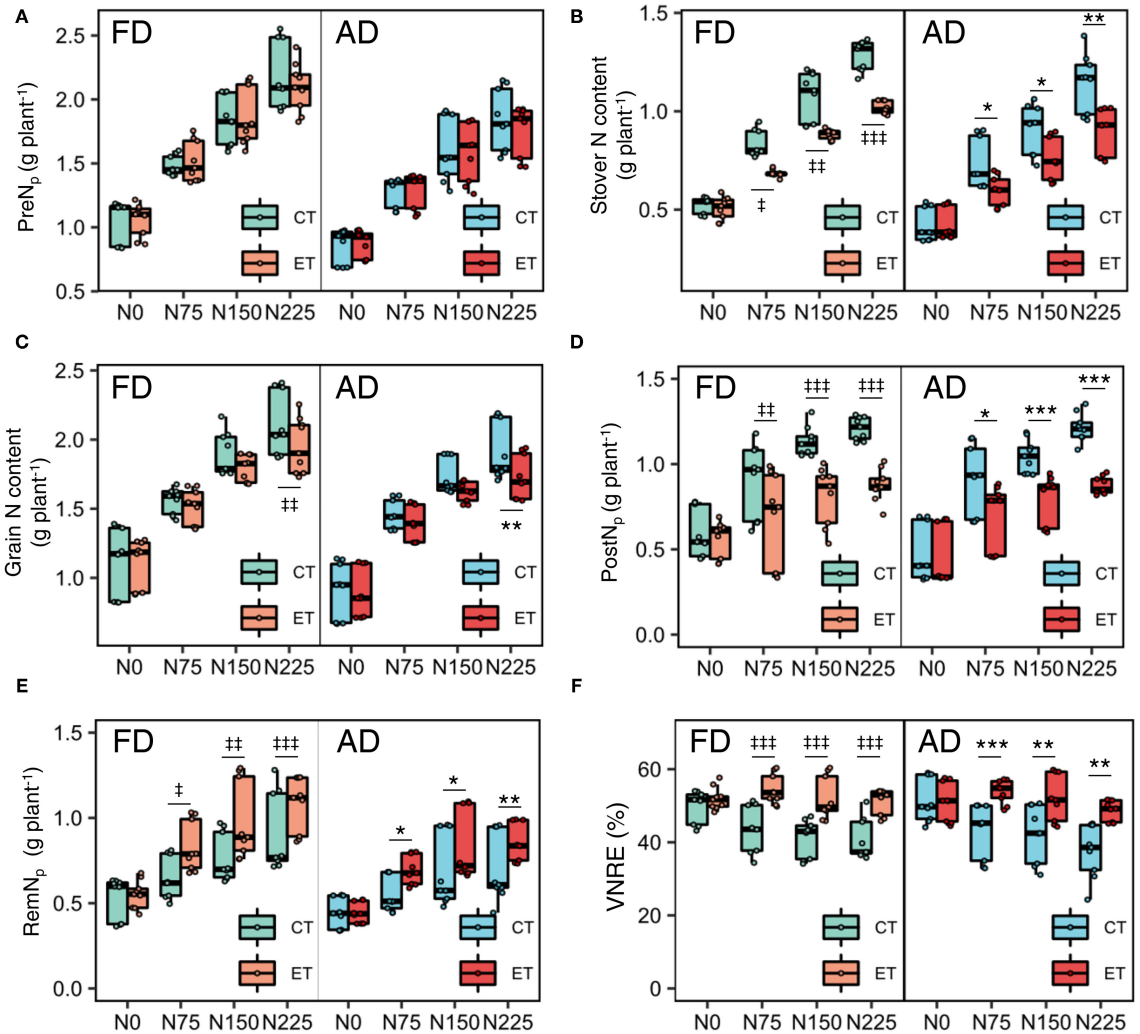
Effects of ethephon on maize pre-silking N uptake per plant [PreN_p_, **(A)**], N content in stover **(B)** and grain **(C)**, post-silking N uptake per plant [PostN_p_, **(D)**], post-silking N remobilization per plant [RemN_p_, **(E)**] and VNRE **(F)** under different N application rates and plant densities. VNRE is vegetative N remobilization efficiency. Data points represent multi-year data under each treatment factor, and the thick lines, lower and upper edges, whiskers outside the boxes represent means, 25th and 75th, 5th and 95th percentiles of all data, respectively. FD is at 6.75 plant m^−2^ and AD is at 7.5 plant m^−2^. N0, N75, N150, N225 are N application rate at 0, 75, 150, 225 kg N ha^−1^ respectively. *(^‡^), **(^‡‡^), ***(^‡‡‡^) means significant difference between CT and ET under AD(FD) determined by Fisher's least significant difference (LSD, *a* = 0.05) at *P*-value for *F*-test < 0.05, < 0.01 and < 0.001, respectively.

Plant N accumulation depends on both the plant biomass and plant N concentration. As shown in Table 2, the whole plant N content per hectare at the silking stage and the N content in the stover and grains per hectare at the harvesting stage all significantly increased with increased plant density in both 2014 and 2017. These parameters increased with increasing N application rates in all three experimental years. In addition, in all three years, the plant N content at the silking stage was not significantly different between the ethephon treatment and control, while the ethephon treatment resulted in significantly decreased N content in both the stover and grain. As with the results for biomass and N concentrations, significant interactions between the ethephon treatment and the N application rate were observed, while the significant interaction between the ethephon treatment and the plant density was rarely observed (Table 2). As shown in Figures 3B,C, increasing N application rate enhanced the effects of ethephon on N content in both the stover and grain, but the effects of ethephon were similar between AD and FD conditions.

**Table 2:** The effects of ethephon, N application rate, and plant density on maize plant N content, N uptake, and remobilization traits at the silking or harvesting stages in 2014, 2015, and 2017.

**Year**	**Treatment**	**N**_**silk**_		**N**_**stover**_		**N**_**grain**_		**NHI**		**PostN**		**RemN**		**VNRE**	
		**(kg ha** ^**−1**^ **)**		**(kg ha** ^**−1**^ **)**		**(kg ha** ^**−1**^ **)**		**(%)**		**(kg ha** ^**−1**^ **)**		**(kg ha** ^**−1**^ **)**		**(%)**	
2014	PGR														
	CT	106.1	a	67.3	a	112.1	a	63.2	b	73.4	a	38.7	b	37.8	b
	ET	105.6	a	54.2	b	109.4	b	67.2	a	58.0	b	51.4	a	48.7	a
	Nitrogen														
	N0	72.9	d	37.4	d	80.0	d	68.2	a	44.5	c	35.5	d	48.7	a
	N75	96.0	c	55.1	c	111.7	c	67.1	b	70.7	b	40.9	c	42.7	b
	N150	117.6	b	69.9	b	120.9	b	63.5	c	73.2	ab	47.7	b	40.6	c
	N225	136.8	a	80.8	a	130.5	a	62.0	d	74.4	a	56.0	a	40.9	c
	Density														
	FD	104.1	b	57.3	b	107.3	b	65.8	a	60.5	b	46.8	a	45.6	a
	AD	107.5	a	64.3	a	114.2	a	64.6	b	70.9	a	43.3	b	40.8	b
	Source of variance (α = 0.05)														
	P	ns		^***^		^*^		^***^		^***^		^***^		^***^	
	N	^***^		^***^		^***^		^***^		^***^		^***^		^***^	
	D	^***^		^***^		^***^		^***^		^***^		^***^		^***^	
	P × N	ns		^***^		ns		^***^		^***^		^***^		^***^	
	P × D	^***^		^*^		ns		^**^		^*^		^**^		ns	
	N × D	^***^		^***^		ns		^**^		ns		^***^		^***^	
	P × N × D	ns		^*^		ns		ns		ns		ns		ns	
2015	PGR														
	CT	93.1	a	51.9	a	104.0	a	67.3	b	62.8	a	41.2	b	45.3	b
	ET	91.9	a	44.9	b	97.2	b	68.6	a	50.2	b	47.0	a	51.6	a
	Nitrogen														
	N0	69.7	d	31.0	d	73.8	d	70.4	a	35.0	d	38.7	d	55.5	a
	N75	85.7	c	43.6	c	100.2	c	69.6	b	58.1	c	42.1	c	49.0	b
	N150	99.5	b	53.5	b	111.6	b	67.5	c	65.6	b	46.0	b	46.1	c
	N225	115.2	a	65.6	a	116.9	a	64.1	d	67.3	a	49.6	a	43.1	d
	Density														
	FD	93.0	a	49.8	a	97.0	b	66.6	a	53.7	a	43.2	b	47.1	b
	AD	92.0	a	47.1	b	104.2	a	69.2	b	59.3	b	44.9	a	49.7	a
	Source of variance (α = 0.05)														
	P	ns		^***^		^***^		^***^		^***^		^***^		^***^	
	N	^***^		^***^		^***^		^***^		^***^		^***^		^***^	
	D	ns		^***^		^***^		^***^		^**^		^*^		^***^	
	P × N	ns		^***^		^***^		^***^		^***^		^***^		^***^	
	P × D	^*^		^*^		^**^		ns		ns		^*^		ns	
	N × D	ns		^***^		^***^		^***^		^***^		^*^		^*^	
	P × N × D	ns		^*^		^**^		^*^		^**^		^*^		^*^	
2017	PGR														
	CT	109.7	a	57.1	a	107.9	a	65.3	b	55.5	a	52.5	b	47.9	b
	ET	108.4	a	47.8	b	99.9	b	67.5	a	39.3	b	60.6	a	55.3	a
	Nitrogen														
	N0	53.0	d	27.0	d	51.8	d	65.8	c	25.8	d	26.0	d	48.9	b
	N75	101.5	c	46.3	c	91.2	c	66.3	b	36.0	c	55.2	c	54.3	a
	N150	133.4	b	61.5	b	127.0	b	67.5	a	55.2	b	71.9	b	53.8	a
	N225	148.3	a	75.2	a	145.7	a	66.1	bc	72.6	a	73.2	a	49.4	b
	Density														
	FD	106.0	b	50.9	b	98.3	b	65.8	b	43.2	b	55.1	a	51.5	a
	AD	112.1	a	54.1	a	109.5	a	66.9	a	51.5	a	58.0	b	51.7	a
	Source of variance (α = 0.05)														
	P	ns		^***^		^***^		^***^		^***^		^***^		^***^	
	N	^***^		^***^		^***^		^***^		^***^		^***^		^***^	
	D	^***^		^***^		^**^		^***^		^***^		^***^		ns	
	P × N	^***^		^***^		^***^		^*^		^***^		^***^		^***^	
	P × D	^**^		ns		ns		^*^		^*^		^***^		^***^	
	N × D	^**^		^*^		^***^		ns		^***^		^***^		^***^	
	P × N × D	^*^		ns		ns		ns		^*^		^***^		^*^	

*CT and ET represented control and ethephon; FD and AD represented farmers' plant density (6.75 plant m^−2^) and advanced plant density (7.5 plant m^−2^); N0, N75, N150, and N225 indicated N application rates at 0, 75, 150, 225 kg N ha^−1^, respectively. N_silk_, plant N content at the silking stage, viz. pre-silking N uptake per plant; N_stover_, plant N content in stover at the harvesting stage; N_grain_, plant N content in grain at harvesting; NHI, N harvest index; PostN, post-silking N uptake; RemN, post-silking N remobilization; VNRE, vegetative N remobilization efficiency. Within ethephon, N application rate or plant density, values followed by the same letters in the same volume indicated no significant difference (P < 0.05). And ns indicated not significant (P > 0.05)*,

*, **, *** *indicated significant at P < 0.05, 0.01 and 0.001, respectively*.

Although the ethephon treatment reduced the N content in both the stover and grain, the ethephon treatment increased the N harvest index (NHI) significantly, which suggests that the ethephon application promoted more N allocation to the grains rather than the stovers. Grain N accumulation during the reproductive period comes from the PostN and RemN. Ethephon applications resulted in substantial decreases in PostN of 20.90, 20.02, and 29.37%, but significantly increases in RemN of 32.64, 14.10, and 15.71% in 2014, 2015 and 2017, respectively (Table 2). Consequently, ethephon improved the VNRE during the growing seasons of all three years. Moreover, significant ethephon × N application rate and ethephon × plant density interactive effects on PostN, RemN, and NHI were observed in all three seasons, although no significant ethephon × plant density interactive effects on PostN and NHI was observed during 2015. N applications significantly enhanced the effects of ethephon on PostN and RemN compared with the N0 treatment. Similar trends in the effects of ethephon on PostN were observed between FD and AD conditions, while the difference between ET and CT was higher under FD than that under AD conditions with consistent rates of N application. Significant differences in VNRE were observed between CT and ET under N applied treatments, but the VNRE with ET at N0 was not significantly different from CT under both AD and FD conditions (Figure 3F).

### Grain Yield and Its Components

**Figure 4:**
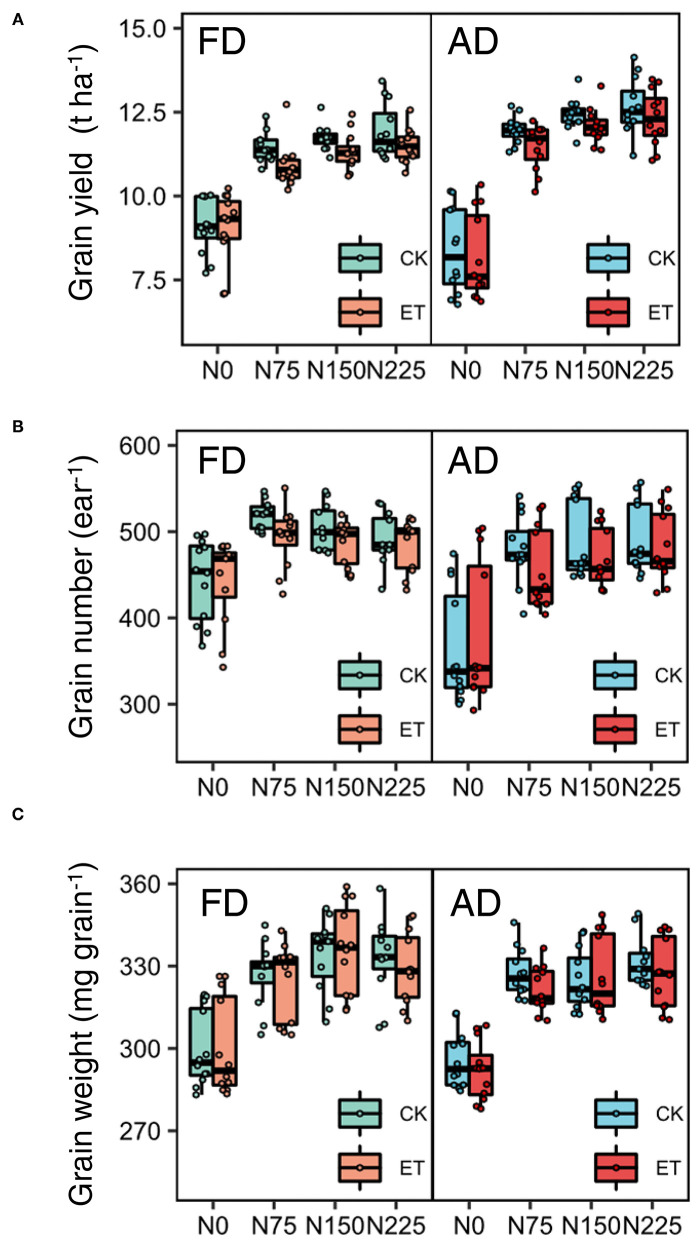
Effects of ethephon on maize grain yield **(A)**, grain number **(B)**, and grain weight **(C)** under different N application rates and densities. Data points represent multi-year data under each treatment factor, and the thick lines, lower and upper edges, whiskers outside the boxes represent means, 25th and 75th, 5th and 95th percentiles of all data, respectively. FD is at 6.75 plant m^−2^ and AD is at 7.5 plant m^−2^. N0, N75, N150, N225 were N application rates at 0, 75, 150, 225 kg N ha^−1^ respectively.

**Figure 5:**
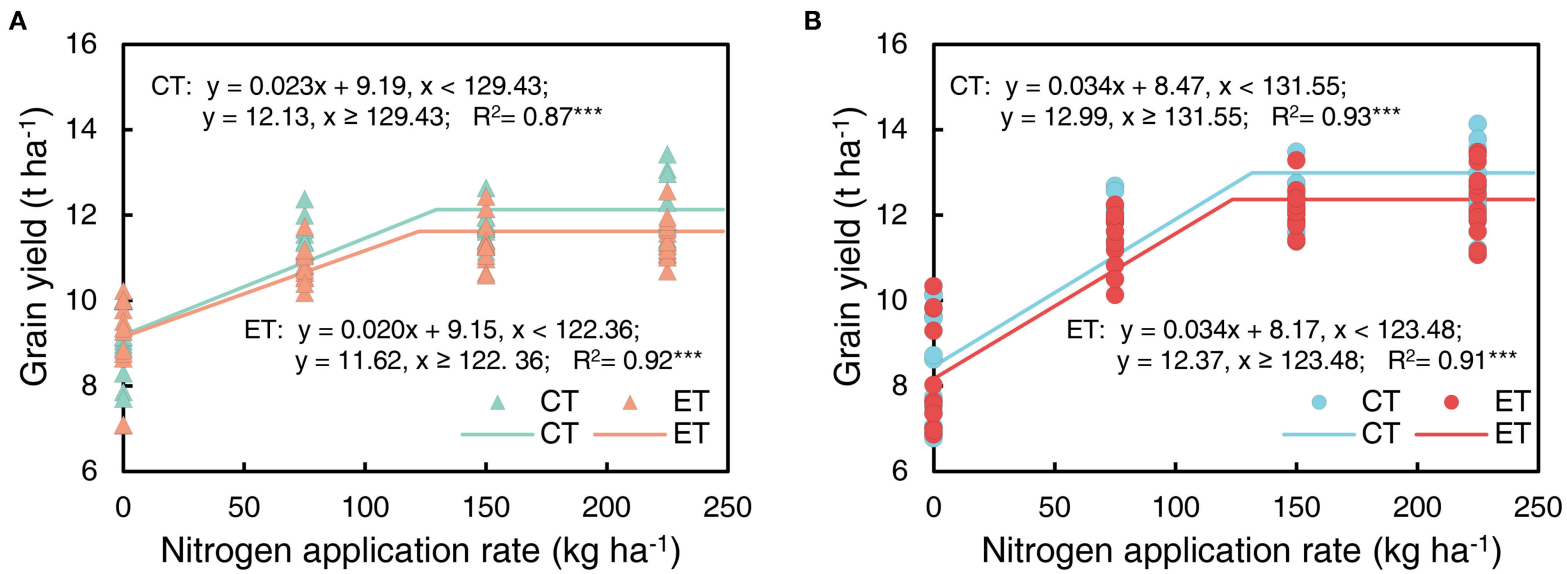
Maize grain yield as a function of increasing N application rate in ethephon treated maize (ET) and control (CT) under farmer's plant density [**(A)**, FD 6.75 plant m^−2^] and advanced plant density [**(B)**, AD 7.5 plant m^−2^]. The relationship between the N application rate and grain yield was simulated using the “linear + plateau” model in the SAS program. Data points represented multi-year data under each treatment factor; The regression model and R^2^ were performed in each panel. *** means the significance of correlation coefficient (R) at *P*-value for *F*-test < 0.001.

Grain yield decreased with the ethephon treatments but increased with increasing N application rates and plant density. Similar trends were observed across the growing seasons in all three years (Table 3). Overall, increases in the grain yield were observed under AD conditions owing to the higher ear number compared with FD, while the effects of plant density on grain weight and grain number were consistent during separate years. N applications of N75 to N225 significantly increased the yield by 14.40 to 64.22% compared with N0 treatments in all three years, however, the difference in yield was not significant between N150 and N225 in either the 2014 or the 2015 growing seasons. The effects of the N application rate on grain numbers and grain weight showed similar trends to that of yield. The ethephon applications significantly decreased grain numbers by 2.79 and 5.04% in 2015 and 2017, respectively, and grain weight by 1.05 and 1.25% in 2014 and 2015, respectively. In addition, significant interactions between the N application rate and plant density were observed in the effects on grain yield, however, there were no significant interactions between the ethephon treatments and N application rate, or between the ethephon treatments and density on grain yield in any of the three years. However, as shown in Figure 4, ethephon applications had a greater effect on yield under any rate of N application than that under the N0 condition. Although the ethephon application decreased the yield under each treatment, the yield with ethephon under AD conditions was still higher than the yield with ethephon or control under FD conditions. Figure 5 shows the “linear + plateau” model simulation of the relationship between grain yield and N application rate. Across the three years, the optimal N application rate required to achieve the maximum grain yield under FD conditions was found to be 129.43 kg ha^−1^ and 122.36 kg ha^−1^ for the control and the ethephon treated maize, respectively. Similar trends were observed under AD conditions, with values of 131.55 kg ha^−1^ and 123.48 kg ha^−1^ for the control and the ethephon treated maize. Also, ethephon treatment and increasing plant density both had significant positive effects on the harvest index.

**Table 3:** The effects of ethephon, N application rate, and plant density on maize yield components, yield, and harvest index in 2014, 2015, and 2017.

**Year**	**Treatment**	**Grain yield**		**Grain number**		**1000-grain**		**Harvest index**	
		**(t ha** ^**−1**^ **)**		**(ear** ^**−1**^ **)**		**weight (g)**		**(%)**	
2014									
	PGR								
	CT	11.3	a	499.9	a	322.7	a	53.6	b
	ET	11.1	b	502.6	a	319.3	b	55.1	a
	Nitrogen								
	N0	9.9	c	466.3	b	306.4	b	53.4	b
	N75	11.4	b	510.9	a	325.6	a	55.2	a
	N150	11.7	a	513.6	a	326.3	a	54.6	ab
	N225	11.7	a	514.1	a	325.6	a	54.2	ab
	Density								
	FD	10.9	b	489.0	b	330.6	a	54.0	a
	AD	11.4	a	513.5	a	311.4	b	54.7	a
	Source of variance (α = 0.05)								
	P	^**^		ns		^***^		^**^	
	N	^***^		^***^		^***^		ns	
	D	^***^		^***^		^***^		ns	
	P × N	ns		^***^		^***^		ns	
	P × D	ns		ns		^*^		ns	
	N × D	^***^		^***^		^***^		^**^	
	P × N × D	ns		^*^		^*^		ns	
2015									
	PGR								
	CT	11.1	a	471.8	a	315.8	a	54.0	a
	ET	10.5	b	458.6	b	311.9	b	54.0	a
	Nitrogen								
	N0	8.3	c	405.9	b	297.8	c	53.2	b
	N75	11.2	b	480.2	a	314.9	b	54.1	ab
	N150	11.8	a	488.7	a	319.4	ab	54.8	a
	N225	11.9	a	486.0	a	323.4	a	53.9	ab
	Density								
	FD	10.8	a	511.5	a	309.3	b	52.9	b
	AD	10.8	a	418.9	b	318.4	a	55.1	a
	Source of variance (α = 0.05)								
	P	^***^		^**^		^*^		ns	
	N	^***^		^***^		^***^		^*^	
	D	ns		^***^		^***^		^***^	
	P × N	ns		ns		ns		ns	
	P × D	ns		ns		ns		ns	
	N × D	^***^		^***^		ns		^***^	
	P × N × D	ns		ns		ns		ns	
2017									
	PGR								
	CT	11.2	a	446.2	a	330.4	a	52.3	b
	ET	10.8	b	423.7	b	327.6	a	54.0	a
	Nitrogen								
	N0	7.8	c	351.7	b	286.7	c	51.0	b
	N75	11.8	b	468.3	a	335.1	b	53.9	a
	N150	12.2	b	458.1	a	346.9	a	54.1	a
	N225	12.8	a	461.7	a	347.2	a	53.5	a
	Density								
	FD	10.9	b	449.0	a	331.6	a	51.9	b
	AD	11.3	a	420.8	b	326.4	b	54.4	a
	Source of variance (α = 0.05)								
	P	^*^		^***^		ns		^**^	
	N	^***^		^***^		^***^		^***^	
	D	^*^		^***^		^*^		^***^	
	P × N	ns		^*^		ns		ns	
	P × D	ns		ns		ns		ns	
	N × D	^**^		^***^		ns		ns	
	P × N × D	ns		ns		ns		ns	

*CT and ET represented control and ethephon treatment; FD and AD represented farmers' plant density (6.75 plant m^−2^) and advanced plant density (7.5 plant m^−2^). N0, N75, N150, and N225 indicated N application rates at 0, 75, 150, 225 kg N ha^−1^, respectively. Within ethephon, N application rate or plant density, values followed by the same letters in the same volume indicated no significant difference (P < 0.05). And ns indicated not significant (P > 0.05)*,

*, **, *** *indicated significant at P < 0.05, 0.01, and 0.001, respectively*.

### Nitrogen Use Efficiency Indices

**Figure 6:**
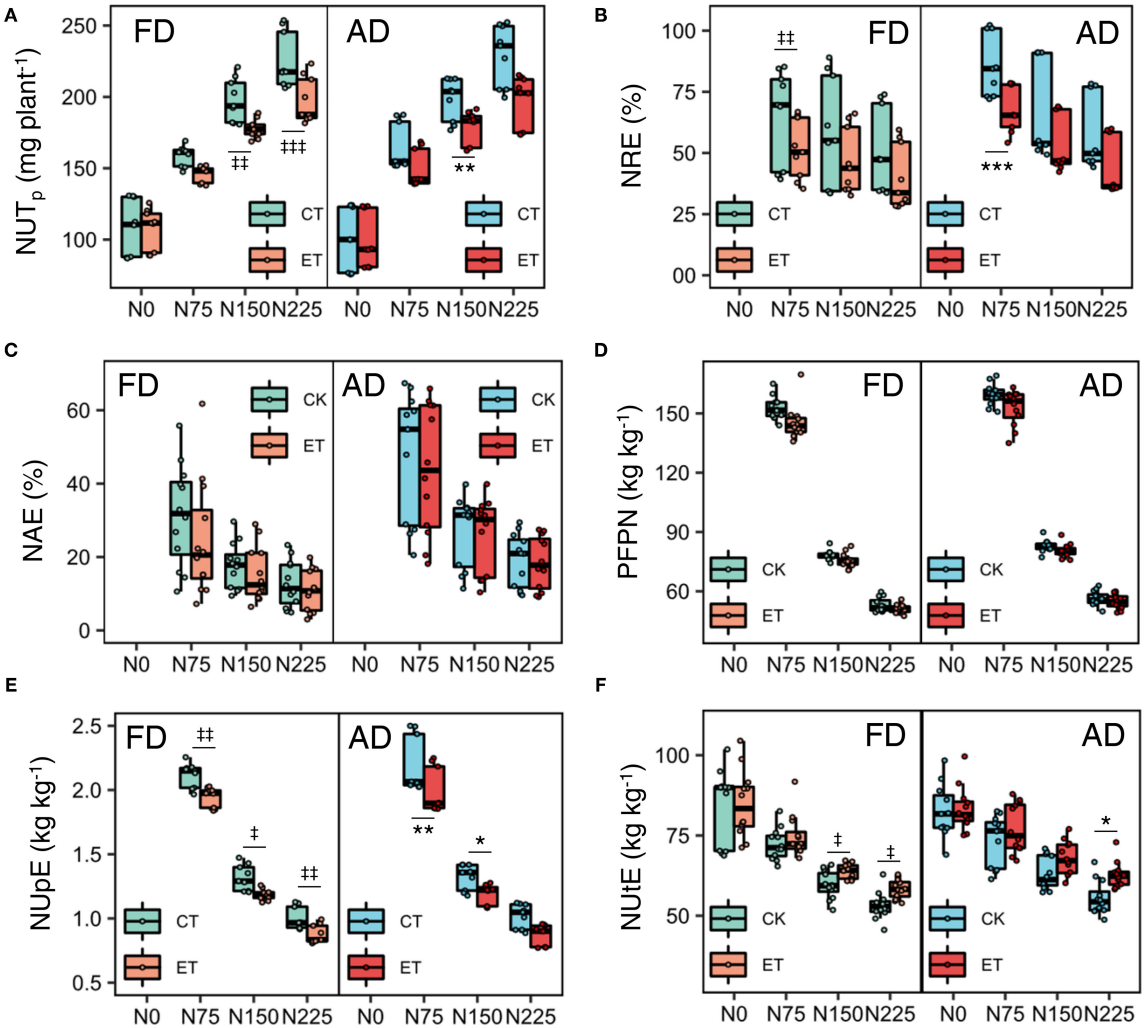
Effects of ethephon on total N uptake per plant **(A)**, NRE **(B)**, NAE **(C)**, PFPN **(D)**, NUpE **(E)**, and NUtE **(F)** under different N application rates and plant densities. NUT_p_ is total N uptake per plant; NRE, N recovery efficiency; NAE, N agronomic efficiency; PFPN, partial factor productivity of N; NUpE, N uptake efficiency; NIE, N internal efficiency. Data points represent multi-year data under each treatment factor, and the thick lines, lower and upper edges, whiskers outside the boxes represent means, 25th and 75th, 5th and 95th percentiles of all data, respectively. FD is at 6.75 plant m^−2^and AD is at 7.5 plant m^−2^. N0, N75, N150, N225 are N application rate at 0, 75, 150, 225 kg N ha^−1^ respectively. *(^‡^), **(^‡‡^), ***(^‡‡‡^) means significant difference between CT and ET under AD(FD) determined by Fisher's least significant difference (LSD, *a* = 0.05) at *P*-value for *F*-test < 0.05, < 0.01 and < 0.001, respectively.

During the three growing seasons of the experimental years, total and single plant N uptake (NUT) was significantly influenced by ethephon, N application rate, and plant density (Table 4 and Figure 6). Specifically, NUT improved with increasing N application rates and plant density, while ethephon applications markedly decreased NUT by 8.80, 8.83, and 10.55% in 2014, 2015, and 2017, respectively. The ethephon application significantly decreased NRE by 11.53–26.29%, NAE by 1.56–19.72%, PFPN by 1.81–7.35%, and NUpE by 8.44–11.31% respectively in the three years. On the contrary, ethephon significantly increased NUtE by 5.89, 3.03, and 5.28% compared with control in 2014, 2015, and 2017 respectively. Furthermore, significant interactive effects of ethephon × N interactions were observed over the course of the growing seasons on NUT, NRE, NUpE, and NUtE. The positive effects of ethephon on NUtE were larger under conditions with medium and high N application rates (N150 and N225) than low N application rates (N0 and N75, Figure 6F).

**Table 4:** The effects of ethephon, N application rate, and plant density on maize N use efficiency indices in 2014, 2015, and 2017.

**Year**	**Treatment**	**NUT**		**NRE**		**NAE**		**PFPN**		**NUpE**		**NUtE**	
		**(kg ha** ^**−1**^ **)**		**(kg kg** ^**−1**^ **)**		**(%)**		**(kg kg** ^**−1**^ **)**		**(kg kg** ^**−1**^ **)**		**(kg kg** ^**−1**^ **)**	
2014													
	PGR												
	CT	179.4	a	60.0	a	14.0	a	94.9	a	1.56	a	65.6	b
	ET	163.6	b	44.2	b	11.9	b	92.6	b	1.40	b	69.5	a
	Nitrogen												
	N0	117.4	d									84.8	a
	N75	166.8	c	65.8	a	19.1	a	151.4	a	2.22	a	68.3	b
	N150	190.7	b	48.9	b	11.9	b	78.0	b	1.27	b	61.5	c
	N225	211.3	a	41.7	c	7.8	c	51.9	c	0.94	c	55.5	d
	Density												
	FD	164.6	b	50.7	b	9.5	b	91.0	b	1.41	b	69.2	a
	AD	178.5	a	53.6	a	16.3	a	96.5	a	1.54	a	65.8	b
	Source of variance (a = 0.05)												
	P	^***^		^***^		^*^		^**^		^***^		^***^	
	N	^***^		^***^		^***^		^***^		^***^		^***^	
	D	^***^		^**^		^***^		^***^		^***^		^***^	
	P × N	^***^		^***^		ns		ns		^***^		^***^	
	P × D	ns		ns		ns		ns		ns		ns	
	N × D	^*^		^**^		^**^		^*^		^***^		^***^	
	P × N × D	ns		ns		ns		ns		ns		ns	
2015	PGR												
	CT	155.9	a	44.9	a	28.7	a	97.2	a	1.33	a	68.6	b
	ET	142.1	b	39.7	b	23.1	b	90.0	b	1.22	b	70.7	a
	Nitrogen												
	N0	104.7	d									75.2	a
	N75	143.8	c	52.2	a	38.8	a	149.6	a	1.92	a	73.9	a
	N150	165.1	b	40.3	b	23.1	b	78.5	b	1.10	b	67.7	b
	N225	182.5	a	34.6	c	15.8	c	52.7	c	0.81	c	61.8	c
	Density												
	FD	146.7	b	31.4	b	17.6	b	91.4	b	1.23	a	66.6	b
	AD	151.3	a	53.3	a	34.3	a	95.8	a	1.32	b	72.7	a
	Source of variance (a = 0.05)												
	P	^***^		^***^		^***^		^***^		^***^		^*^	
	N	^***^		^***^		^***^		^***^		^***^		^***^	
	D	^***^		^***^		^***^		^**^		^***^		^***^	
	P × N	^***^		^***^		^*^		^**^		^***^		ns	
	P × D	^**^		^***^		ns		ns		^***^		ns	
	N × D	^***^		^***^		^***^		ns		^***^		ns	
	P × N × D	^**^		ns		ns		ns		ns		ns	
2017	PGR												
	CT	165.2	a	81.0	a	35.2	a	99.2	a	1.44	a	70.9	b
	ET	147.7	b	62.1	b	34.7	a	97.4	a	1.28	b	74.7	a
	Nitrogen												
	N0	78.8	d									93.3	a
	N75	137.5	c	78.2	a	53.1	a	156.7	a	1.83	a	81.1	b
	N150	188.6	b	73.2	b	29.6	b	81.5	b	1.26	b	61.7	c
	N225	220.9	a	63.2	c	22.2	c	56.7	c	0.98	c	55.0	d
	Density												
	FD	149.3	b	63.7	b	29.2	b	95.5	b	1.28	b	71.0	b
	AD	163.6	a	79.3	a	40.7	a	101.1	a	1.43	a	74.6	a
	Source of variance (a = 0.05)												
	P	^***^		^***^		ns		ns		^***^		^*^	
	N	^***^		^***^		^***^		^***^		^***^		^***^	
	D	^***^		^***^		^***^		^**^		^**^		^*^	
	P × N	^***^		^**^		ns		ns		^*^		^**^	
	P × D	ns		^*^		ns		ns		ns		ns	
	N × D	^***^		^***^		ns		ns		ns		ns	
	P × N × D	ns		ns		ns		ns		ns		ns	

*CT and ET represented control and ethephon; FD and AD represented farmers' plant density (6.75 plant m^−2^) and advanced plant density (7.5 plant m^−2^); N0, N75, N150, and N225 indicated N application rates at 0, 75, 150, 225 kg N ha^−1^, respectively. NUT, total N uptake; NRE, N recovery efficiency; NAE, N agronomic efficiency; PFPN, partial factor productivity of N; NUpE, N uptake efficiency; NUtE, N utilization efficiency. Within ethephon, N application rate or plant density, values followed by the same letters in the same volume indicated no significant difference (P < 0.05). And ns indicated not significant (P > 0.05)*,

*, **, *** *indicated significant at P < 0.05, 0.01 and 0.001, respectively*.

### Nitrate and Ammonium Fluxes in Xylem Bleeding Sap

**Figure 7:**
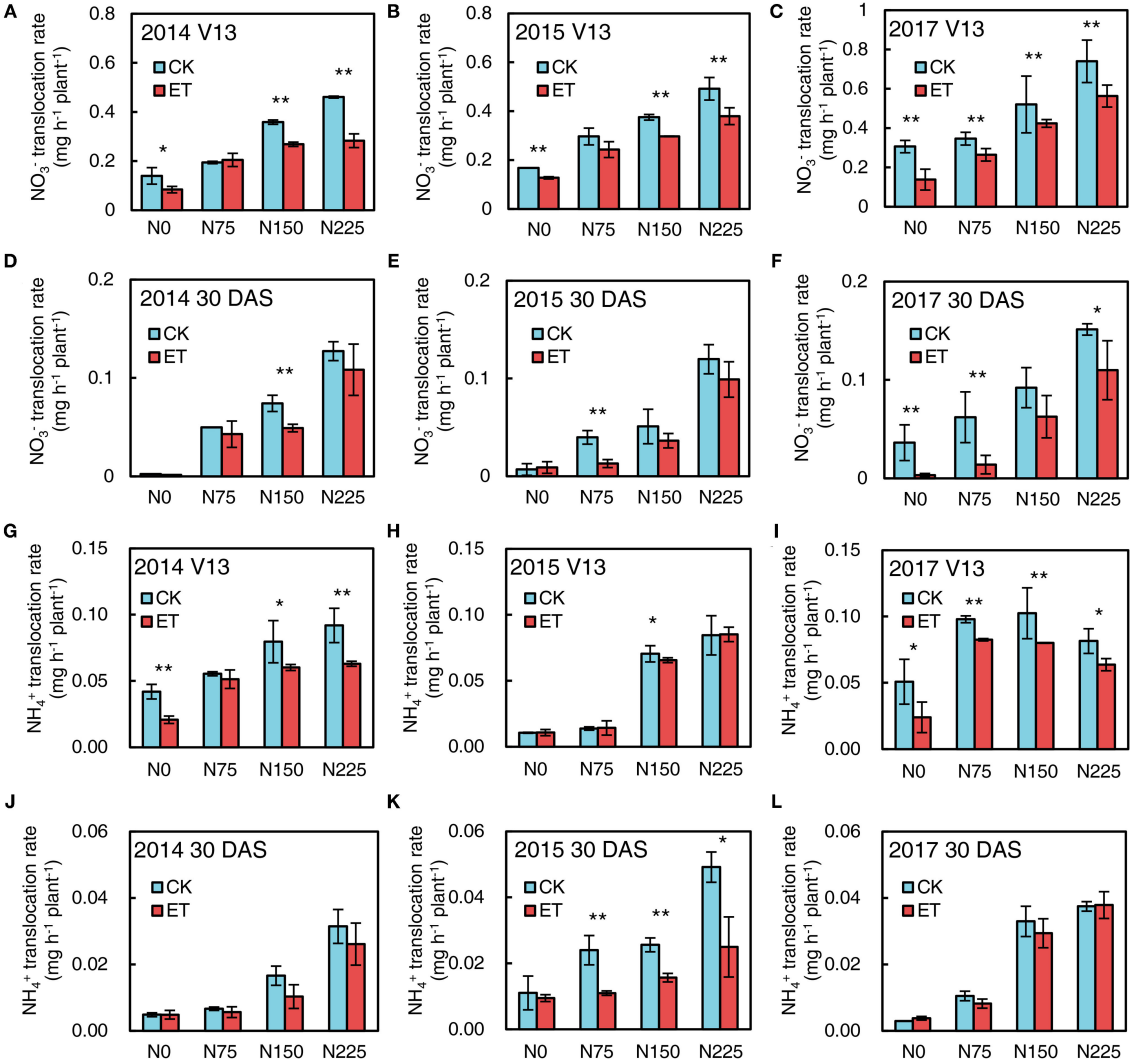
Effects of ethephon on maize ${\text{NO}}_{3}^{-}$ and ${\text{NH}}_{4}^{+}$ fluxes in xylem bleeding sap at advanced plant density under four N application rates at V13 and 30 DAS in 2014, 2015, and 2017. **(A–C)** exhibited ${\text{NO}}_{3}^{-}$ fluxes at V13 in 2014, 2015, and 2017; **(D–F)** exhibited ${\text{NO}}_{3}^{-}$ fluxes at 30 DAS in 2014, 2015, and 2017; **(G–I)** exhibited and ${\text{NH}}_{4}^{+}$ fluxes at V13 in 2014, 2015 and 2017; **(J–L)** exhibited and ${\text{NH}}_{4}^{+}$ fluxes at 30 DAS in 2014, 2015 and 2017, respectively. CT and ET represented control and ethephon treatment; N0, N75, N150, N225 were N application rate at 0, 75, 150, 225 kg N ha^−1^ respectively. Vertical error bars indicate standard deviation. * and ** means significant difference between CT and ET determined by Fisher's least significant difference (LSD, *P* < 0.05) at *P*-value for *F-*test < 0.05 and < 0.01, respectively.

As shown in Figure 7, the ${\text{NO}}_{3}^{-}$ fluxes were much higher than ${\text{NH}}_{4}^{+}$ fluxes in the maize xylem bleeding sap. Increased N application rates significantly increased both ${\text{NO}}_{3}^{-}$ and ${\text{NH}}_{4}^{+}$ flux in xylem bleeding sap at both the V13 stage and 30 days after silking (DAS) in 2014, 2015, and 2017, and the ${\text{NO}}_{3}^{-}$ and ${\text{NH}}_{4}^{+}$ fluxes were much higher at the V13 stage than the silking stage and 30 DAS. Ethephon applications markedly decreased ${\text{NO}}_{3}^{-}$ flux compared with control at the V13 stage and 30 DAS in all three years (Figures 7A–F). Meanwhile, ethephon-treated plants had lower ${\text{NH}}_{4}^{+}$ flux values than control at the V13 stage across all N application rates (Figures 7G–I). Similar negative effects of ethephon on flux occurred at 30 DAS in all three experimental years (Figures 7J–L).

### The Relationship of Grain Yield and N Content With Biomass Accumulation and Nitrogen Uptake and Remobilization

**Figure 8:**
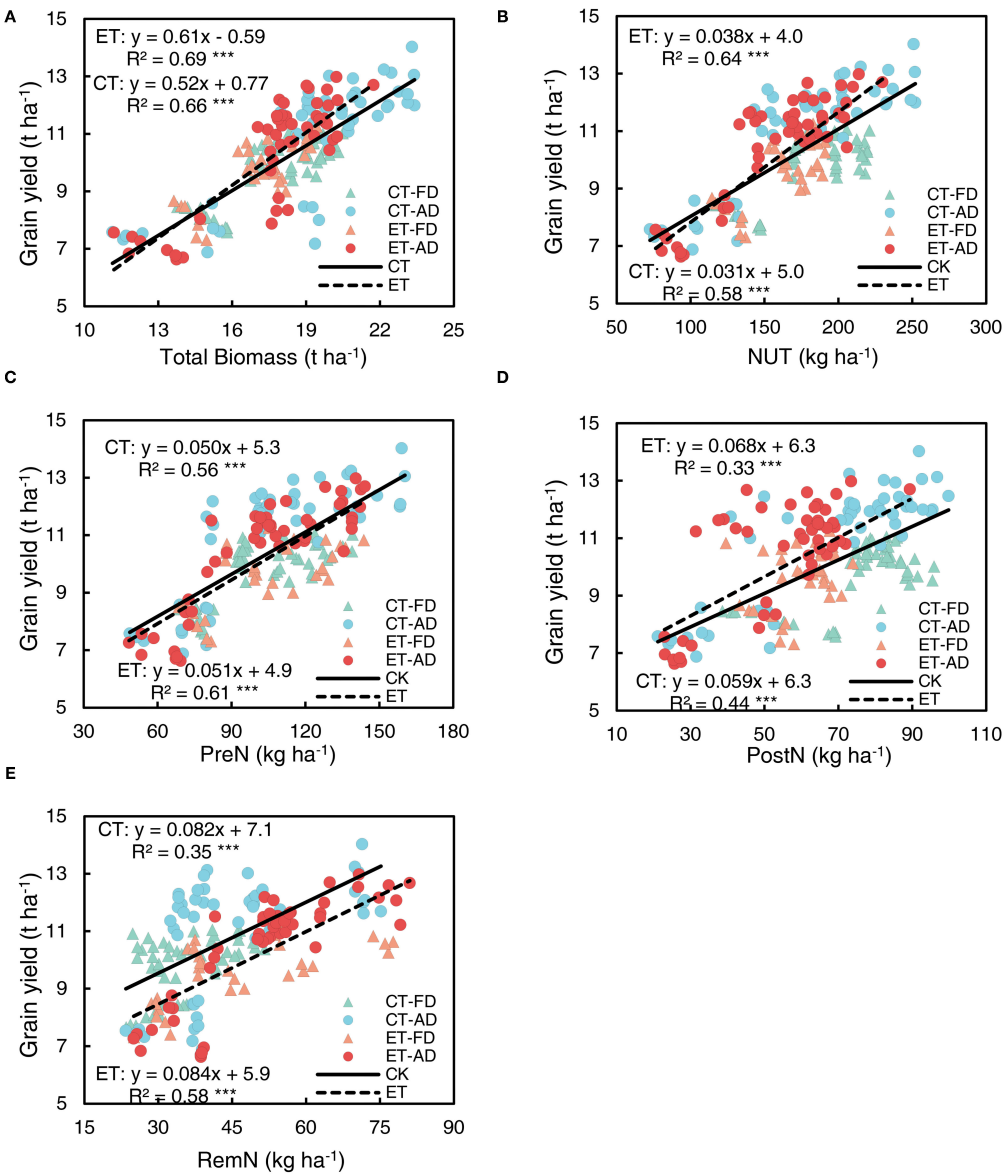
Relationships of grain yield with biomass accumulation, N uptake, and remobilization. **(A)** grain yield vs. total biomass achieved at harvesting. **(B)** grain yield vs. total N uptake (NUT) achieved at harvesting. **(C)** grain yield vs. pre-silking N uptake (PreN). **(D)** grain yield vs. post-silking N uptake (PostN). **(E)** grain yield vs. post-silking N remobilization (RemN). CT and ET represent control and ethephon treatment; Triangle symbols indicate FD (6.75 plant m^−2^), and circle symbols indicate AD (7.5 plant m^−2^). The solid linear regression lines represent the data from control plants that responded to, N application rate, and plant density, while the dashed linear regression lines represent the data from ethephon-treated plants. *** indicate the significance of correlation coefficient (R) at *P* < 0.001.

To investigate the effects of ethephon on the relationship between grain yield, N use, and plant biomass, data on different N application rates and plant densities were combined in correlation and regression analyses. A tight linkage was observed between grain yield and plant biomass at the harvesting stage in both the ethephon treated plants and non-treated control (Figure 8A); the slope associated with this relationship was 25% steeper in the ethephon-treated plants compare with the control (Supplementary Table S1). Grain yield was significantly positively correlated with NUT; the slope associated with this correlation was significantly higher in the ethephon-treated plants than in the control (Figure 8B, Supplementary Table S1). Moreover, grain yield was significantly positively correlated with PreN, PostN and RemN (Figures 8C-E), the associated R^2^ values for these correlations were as follows: PreN (${\text{R}}_{\text{ET}}^{2}$ = 0.56, ${\text{R}}_{\text{CT}}^{2}$ = 0.61) > RemN (${\text{R}}_{\text{ET}}^{2}$ = 0.35, ${\text{R}}_{\text{CT}}^{2}$ = 0.58) > PostN (${\text{R}}_{\text{ET}}^{2}$ = 0.33, ${\text{R}}_{\text{CT}}^{2}$ = 0.44). The ethephon treatment had little impact on the slopes associated with these relationships (Supplementary Table S1).

**Figure 9:**
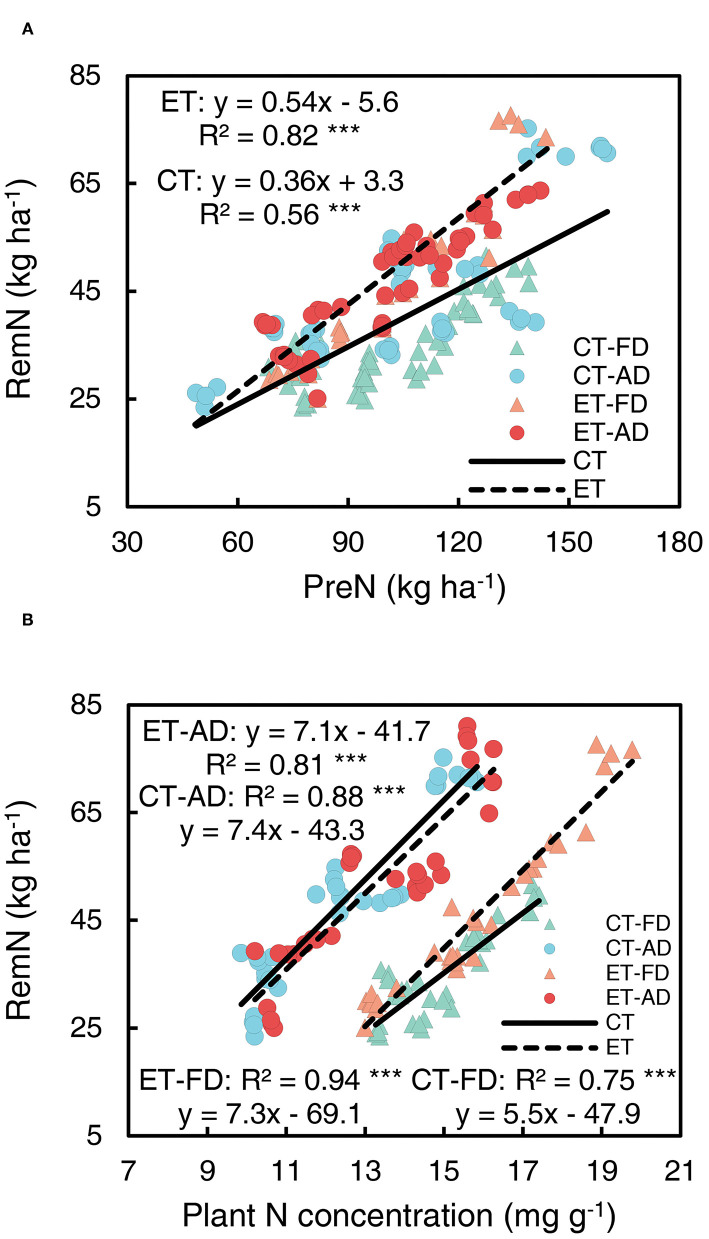
Relationships of post-silking N remobilization with pre-silking N uptake (PreN) **(A)** Plant N concentration **(B)** CT and ET represent control and ethephon treatment, respectively. Triangle symbols indicate farmers' plant 948 density (FD, 6.75 plant m^−2^), and circle symbols indicate advanced plant density (AD, 7.5 plant m^−2^). The solid linear regression lines represent the data from control plants response to, N application rate and plant density, while the dashed linear regression lines represented the data from ethephon-treated plants. ***indicate the significance of correlation coefficient (R) at *P* < 0.001.

Significant correlations were shown between RemN and PreN and between RemN and pre-silking N concentrations (Figure 9). The intercept of the RemN-pre-silking N concentration line was significantly affected by plant density, owing to a reduction in plant N concentration under AD compared with FD conditions. Ethephon markedly increased the slopes of the RemN-PreN regression line (Figure 9A, Supplementary Table S1) and that of the RemN-pre-silking N concentration regression line under FD conditions (Figure 9B, Supplementary Table S1), indicating that ethephon enhanced N remobilization from the stovers to the grains.

**Figure 10:**
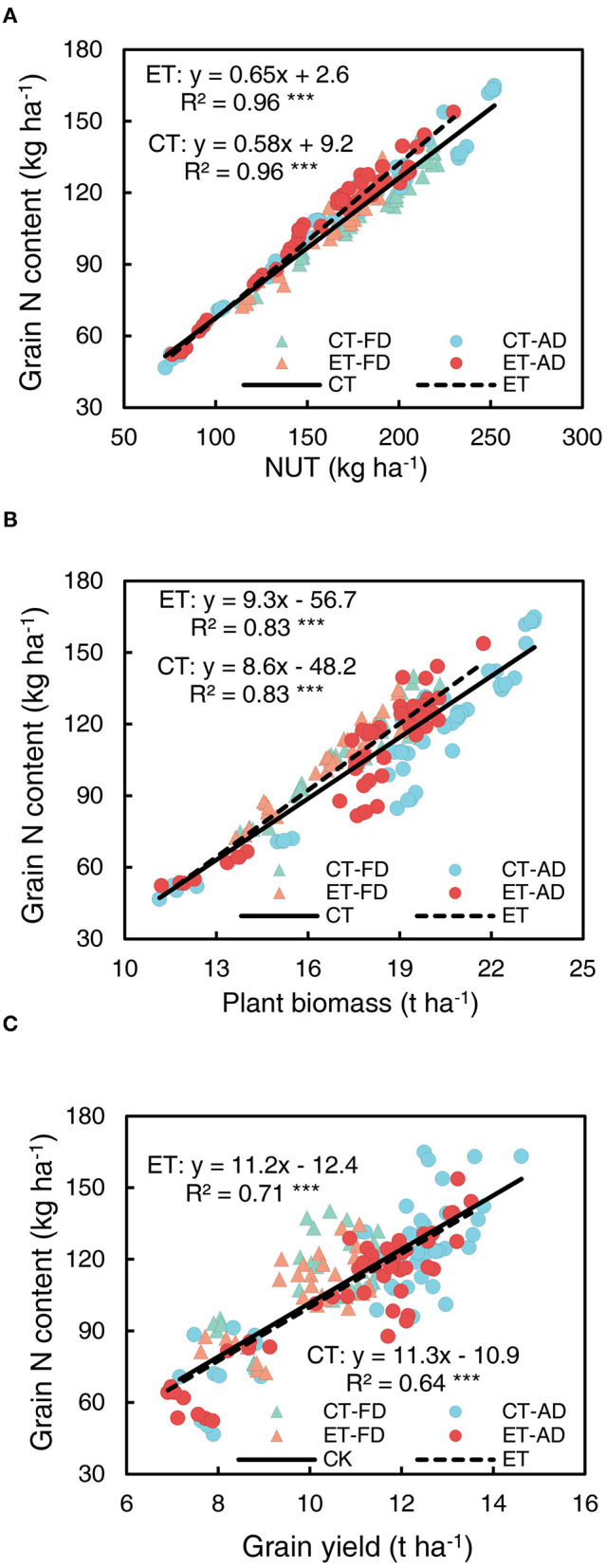
Relationships of grain N content with N uptake (NUT) **(A)**, plant biomass **(B)**, and grain yield **(C)**. CT and ET represented control and ethephon treatment, respectively. Triangle symbols indicate FD (6.75 plant m^−2^), and circle symbols indicate AD (7.5 plant m^−2^). The solid linear regression lines represent the data from control plants' response to, N application rate, and plant density, while the dashed linear regression lines represented the data from ethephon-treated plants. *** indicate the significance of correlation coefficient (R) at *P* < 0.001.

Grain N content had the strongest correlation with NUT (all R^2^ > 95, Figure 10A). Ethephon significantly increased the slope of the regression line for NUT and grain N content (Supplementary Table S1), indicating that ethephon increased the grain N allocation from total N uptake. In addition, grain N content was significantly and strongly correlated with both plant biomass and grain yield (Figures 10B,C). The slope of the regression line for plant biomass and grain N content was steeper in the ethephon-treated maize compared with control, however, the difference between the slopes was not significant. The effects of ethephon on the relationship between the grain yield and N content were also small.

**Figure 11:**
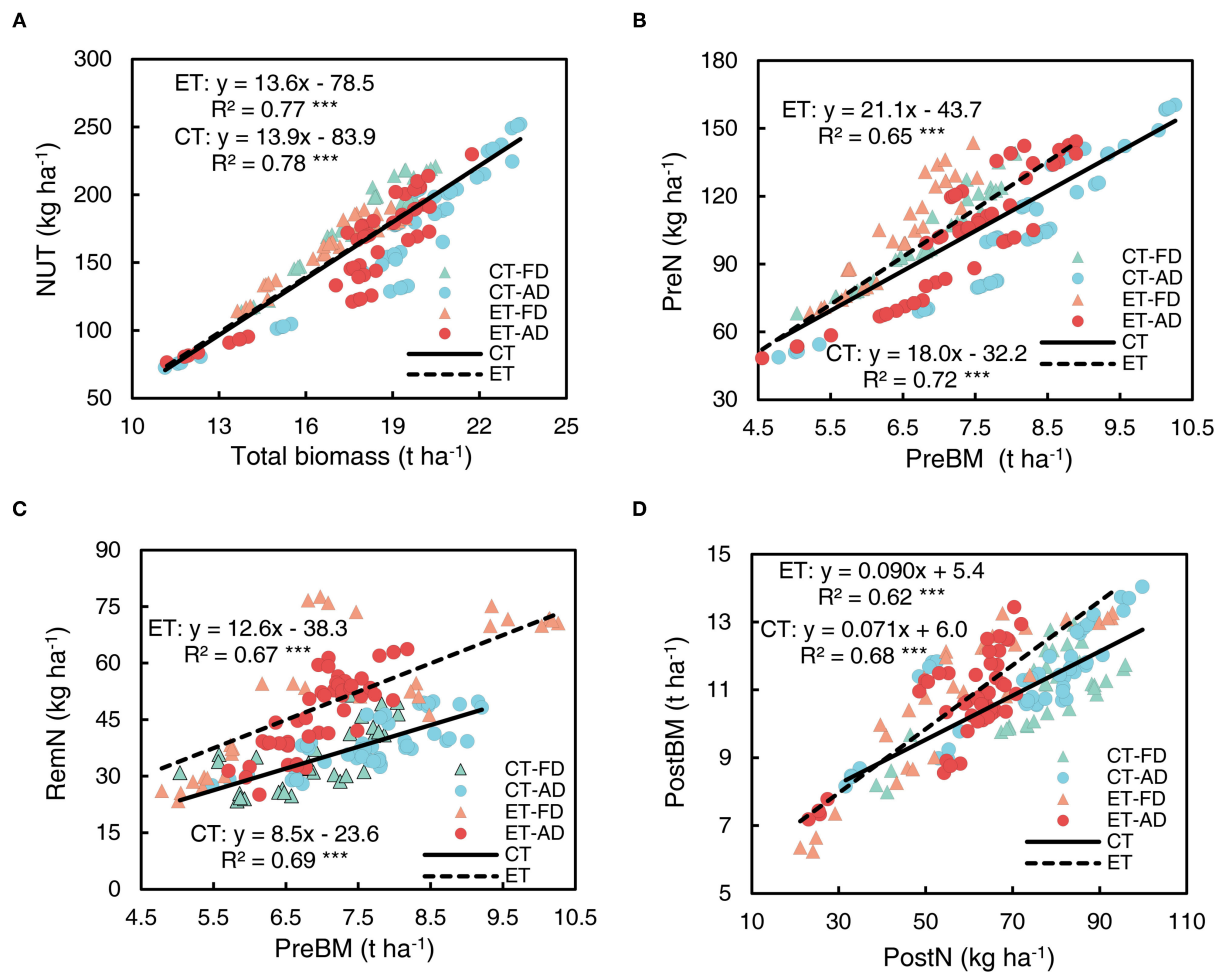
Relationships among N uptake, remobilization, and plant biomass at the different growth stages. **(A)** total N uptake vs. total plant biomass; **(B)** pre-silking N uptake vs. pre-silking biomass (PreBM); **(C)** post-silking N remobilization (RemN) vs. PreBM; **(D)** post-silking biomass vs. post-silking N uptake (PostN). Triangle symbols indicate FD (6.75 plant m^−2^) and circle symbols indicate AD (7.5 plant m^−2^). The solid linear regression lines represent the data from control plants' response to ethephon, N application rate, and plant density, while the dashed linear regression lines represent the data from ethephon-treated plants. *** indicate the significance of correlation coefficient (R) at *P* < 0.001.

NUT was strongly correlated with plant biomass, the effect of ethephon on this positive relationship was small (Figure 11A). Similarly, PreN was significantly correlated with pre-silking biomass (Figure 11B). The correlation between RemN and pre-silking biomass had a R^2^ value to that of the correlation between PreN and pre-silking biomass (Figure 11C). The slope associated with the PreN-pre-silking biomass correlation was not significantly increased by ethephon, but ethephon greatly increased the slope of RemN-pre-silking biomass correlation (Supplementary Table S1), indicating a positive effect of ethephon on RemN. Moreover, the biomass achieved after silking was significantly positively correlated with PostN (Figure 11D), and ethephon significantly increased the slope of this correlation, indicating that high post-silking plant N productivity was achieved with ethephon.

## Discussion

Increasing plant density and N application rates have been shown to effectively increase plant biomass per unit area and yield of maize, however, excessive N application and plant densities decrease NUE, increasing the risk of lodgings and resulting in environmental pollution (Guo et al., 2010; Ju and Christie, 2011). Ethephon application is essential for intensive maize production under conditions of high plant density and N application in order to reduce the risk of lodging. However, rare information about how ethephon regulates maize N use limited the use of chemical control technologies to improve the efficiency of maize production. In this study, field experiments were conducted to evaluate the effects of ethephon on indicators of maize N use and their relationship with biomass and yield.

### Effects of Ethephon on Maize Biomass, Indicators of N Use, and Their Relationship With Grain Yield

The results of our study were consistent with previous reports that increasing N application significantly enhances plant growth and grain production by increasing N uptake in maize (Ciampitti and Vyn, 2011, 2012; Chen et al., 2014). We found that ethephon application also dramatically decreased grain number and grain weight. The loss in grain number and grain weight resulted in a lower grain yield per plant after ethephon application. Previous studies have reported similar trends (Zhou et al., 1999; Shekoofa and Emam, 2008). Previous studies have reported that pre-silking biomass and N uptake substantially affect grain number and grain weight thus influencing maize grain yield (D'Andrea et al., 2008; Ciampitti and Vyn, 2012), and the grain yield is positively correlated with whole-plant N levels at the silking stage (Bertin and Gallais, 2000). In agreement with these previous reports, in our study, the grain yield was strongly positively correlated with plant biomass and NUT (Figures 8A,B). However, the ethephon application significantly repressed maize pre-silking biomass, ${\text{NH}}_{4}^{+}$ and ${\text{NO}}_{3}^{-}$ uptake with N application of 75–225 kg ha^−1^ at densities of 6.75 and 7.50 plant m^−2^. Laboratory experiments suggested that ethephon could significantly reduce maize stalk growth rate by inhibiting the internode cell elongation (Zhang et al., 2020a). The inhibition of ethephon on biomass may be due to a similar mechanism. Lower biomass and N uptake before silking stage could limit the development of the young ears and reduce grain number. The decreased grain yield associated with ethephon may therefore result from the lower biomass observed with N application compared with no N application conditions.

In mustard, the ethephon application increases N assimilation and photosynthesis subjected to different levels of N (Khan et al., 2008; Iqbal et al., 2011). Previous studies suggested that the ethephon application reduced the leaf area but improved the SPAD values before silking (Ye, 2015), and increased the N concentration before silking (Table 1). Similar trends in the relationship between the maize biomass and grain yield with the ethephon treatment were reported by Norberg et al. (1988) and Zhou et al. (1999). Besides, the ethephon application markedly increased the grain harvest index (Table 3). It could be safe to suppose that ethephon increased the photosynthesis per unit leaf area, and the reduced vegetive organs biomass may contribute to the allocation of photosynthate to gain. In other words, these results indicated that the ethephon application could improve the potential productivity of biomass and absorbed N. Observing the NUE indices, the NUtE was observed higher under ethephon treatment in this study. Higher NUtE indicated that the plant transformed more N absorbed to grain and formed yield (Coque and Gallais, 2007; Ciampitti and Vyn, 2011). As a senescence-related phytohormone, ethylene has been identified regulating multiple biological processes (Iqbal et al., 2017). Our results suggested that ethephon reduced N uptake after silking and accelerated N remobilization from the stovers to the grain. These may be accompanied by degradation of chloroplast and cell senescence (Coque and Gallais, 2007; Chen et al., 2015). Lower biomass and accelerated leaf senescence could also provide a mechanism for the lower grain weight under ethephon treatment. Li et al. (2018) suggested that the GA signaling pathway could be involved in the coordination between growth and N metabolism. Our previous laboratory experiments also revealed that the GA pathway is involved in ethephon regulated maize internode elongation (Zhang et al., 2020a). In this study, ethylene may play positive roles in photosynthate and N translocation during the post-silking stage. However, the total N uptake was reduced by ethephon. The mechanism of ethephon on the growth-N metabolism coordination needs further study.

### Effects of Ethephon on Grain N Concentration and Its Relationship With RemN, PostN, and PreN

Grain N concentration is important to maintain the assimilation capacity and grain protein content (Barneix, 2007). Grain N concentration is influenced by genotype, N application rate, and plant density, increasing with greater N application rate and decreasing with increased plant density (Ciampitti and Vyn, 2011; Chen et al., 2015; Chen and Vyn, 2017). Similar trends were observed in this study. N application rate. But interestingly, no significant differences in grain N concentration were observed between ethephon treated and control plants in this study. No significant interactions were observed between ethephon treatment and N and between ethephon treatment and plant density. Generally, grain N is derived from RemN and PostN during the maize reproductive stage (Coque and Gallais, 2007; Chen and Vyn, 2017). Our results indicate that activated RemN may mitigate the loss of PostN under ethephon treatment and thus maintain grain N concentration (Table 2 and Figure 3). It is important to understand the effect of ethephon on N uptake and N remobilization to address N deficiencies by using ethephon.

Kamiji et al. (2014) previously suggested that shoot biomass was the driver for N uptake in N-deficient wheat. Physiological research has also revealed that aboveground sink demand drives N uptake in maize (Bloom, 2015). Another previous study has suggested that ACC, the precursor of ethylene, is involved in the repression of leaf growth under high nitrate conditions (Saiz-Fernández et al., 2017). Inhibiting effects of ethephon on maize biomass were observed in this study, which may explain the lower N uptake associated with ethephon treatment. In addition, the exogenous application of ethylene synthesis precursor ACC and inhibitor AVG could upregulate and downregulate nitrate transporters gene expression in *Arabidopsis* (Tian et al., 2009). Wu et al. (2021) found that ACC application could inhibit the expression of *ZmNRT2.1* in maize root under normal ${\text{NO}}_{3}^{-}$ conditions but promote its expression under ${\text{NO}}_{3}^{-}$ deficient conditions. Thus, it needs further research if the lower N uptake under ethephon treatment could have partly resulted from ethylene signal-regulated N transport genes expression. Besides, ethylene is widely accepted as one of the senescence-related phytohormones and is involved in plant programmed cell death and the development of root architectural traits (He et al., 1996; Schneider et al., 2018). Lower root biomass was observed previously in maize under ethephon treatment (Guan, 2014). Therefore, ethephon-reduced N uptake may also result from reduced root growth and promotion of the senescence process.

Previous research has uncovered a strong positive association between RemN and pre-silking plant N content, with high plant N content as a main driver of RemN at the beginning of the grain filling process (Chen et al., 2015). A strong correlation between RemN and PreN and between RemN and pre-silking plant N concentration was observed in this study (Figure 9). The performance was similar between the ethephon treatment and control. These results indicated that the ethephon-improved pre-silking plant N concentration, instead of the total N content, may promote RemN.

### Effects of Ethephon on N Use Efficiency Responding to N Application Rate and Plant Density

Several previous studies have shown that the ethephon application increases photosynthesis and N assimilation in mustard responding to various N application rates (Khan et al., 2008; Iqbal et al., 2011) and that NUE is increased with ethephon (Iqbal et al., 2011 and Iqbal et al., 2012). In contrast, in this study, the ethephon application significantly reduced NAE, NRE, PFPN, and NUpE in maize at the equivalent levels of N application and plant density. Ethephon application significantly decreased plant biomass, which led to lower grain yield and NUT under different N application rates and plant densities. This could be a primary reason for the lower values observed across some of the NUE indices. Although our simulation analysis of grain yield and N application rate indicated that the optimal N application rate to achieve the maximum grain yield was lower in the ethephon treated maize than that in control plants under both plant densities (Figure 5). Thus, the NUE of ethephon treated maize under the same N input conditions is likely to be underestimated compared with the control. Reduced N application rates are therefore likely to increase NUE when applying ethephon in the field.

The maize hybrid Zhengdan958, widely used in the experimental region, is a “stay-green” cultivar with higher PostN and lower RemN (Chen et al., 2015). The “stay-green” trait results in more residual N in the stover (Shanahan et al., 2008; Kosgey et al., 2013); this undesirable effect is enhanced at an increasing N application rate, as observed in the decrease of VNRE in this study. Thus, the “stay-green” trait may lead to in-season N waste, especially when combined with a high N application rate. Ethephon application may improve RemN and lessen these negative effects of the “stay-green” trait.

Previous studies suggested that the lodging rates increase significantly with plant density, and the degree to which ethephon reduces lodging and increases harvested ear number is greater with high than with low plant densities (Zhang et al., 2017, 2019a). Similar trends were observed in this study, but the difference in the lodging rate between ethephon-treated and control plants was not significant at equivalent N application rates and plant densities. Thus, a similar effect of ethephon on grain yield was observed under different plant densities, and there was no significant interaction between ethephon and plant density. In addition, increasing plant density increases competition between plants for nutrients and space, resulting in lower biomass and N content in of each plant (Tokatlidis et al., 2011; Yan et al., 2017). The results of this study are consistent with this, increasing plant density enhanced the inhibiting effects of ethephon on biomass and N content. However, increasing plant density improves the total N uptake per unit area (Ciampitti et al., 2013). The values of grain yield with HI, NHI, NAE, NRE, and PFPN were all higher in ethephon treated plants at the higher plant density in our study compared with both ethephon treated and control plants at lower plant density. Considering the lodging risks, the improved grain yield observed with ethephon application may increase the NAE and PFPN under high lodging risks conditions, such as high plant density, high temperature, and humidity. In other words, high density may be required in order to use ethephon application to increase grain yield and NUE under high N input conditions.

### Prospects of Using Ethephon in Maize Production

Rising temperatures resulting from climate change are likely to accelerate maize growth and reduce stalk quality causing greater lodging risks particularly in intensive production associated with high plant density and N input (Zhang et al., 2020b; Xue et al., 2017). Ethephon applications have a great potential to reduce these lodging risks, thus improving and stabilizing maize yields. This study revealed the advantages of ethephon in increasing NtUE and NHI but also highlighted its negative effects on N uptake. Thus, it is necessary to develop agronomic practices in improving maize N uptake and to mitigate the loss in grain numbers and weight associated with ethephon for better use of this effective plant growth regulator.

One possible means for this is to use ethephon with other agents that stimulate growth. For example, a mixture of diethyl aminoethyl hexanoate and ethephon was previously shown to increase the grain yield of maize (Xu et al., 2017; Zhang et al., 2019a). Dual applications of 2-diethylaminoethyl-3,4-dichlorophenylether (DCPTA) and ethephon have also been shown to improve dry matter accumulation and grain yield in crops (Li et al., 2019). Meanwhile, modification of ethephon may also be feasible and effective: recent research has suggested that N, N-Diethyl-2-hexanoyl oxygen radicals-ethyl amine (2-ethyl chloride) phosphonic acid salt, which contains an ethephon functional group, was able to improve the photosynthesis of canopy and yield (Huang et al., 2021).

N fertilizer management should also be considered in the future use of ethephon in maize production. Recommendations for the appropriate timings of N application to improve crop NUE has dated back to the previous century (Matson et al., 1998). Compared with applying all N fertilizer prior to sowing, splitting and delaying N application may improve both maize NUE and grain yield (Zhou et al., 2017). Qwing to the reduced N uptake with the ethephon treatment, there may be plenty of N left in the soil, which may increase the leakage and waste of N fertilizer. Thus, using split N application strategies, delays in topdressing with N fertilizers at silking, and integrating N application with irrigation practices may increase the NRE with the use of ethephon. Taking advantage of the positive effects of ethephon in reducing the optimal N application rate and increasing NtUE, these practices may improve NUE and reduce N fertilizer input N.

In addition, revealing the regulatory mechanism of ethylene on maize N uptake and remobilization at the physiological and molecular levels may also catalyze the development of novel approaches to achieving both high resource use efficiency and grain yield using plant growth regulators.

## Conclusions

In this study, ethephon application significantly decreased the maize biomass and N content of maize under both high plant density conditions and those commonly used in China, when N was applied. Ethephon markedly increased plant N concentrations at the silking stage and decreased stover N concentrations at the harvesting stage, while grain N concentration was affected to a smaller degree, regardless of plant density or N application rate. Moreover, ethephon application substantially decreased both pre- and post-silking N uptake and increased post-silking N remobilization compared with untreated plants. Thus, ethephon application significantly improved N productivity, with higher HI, NHI, and NUtE than untreated plants. However, decreased biomass and N uptake with ethephon application resulted in yield reduction and consequently poor NAE, NRE, PFPN, and NUpE compared with untreated plants. When applying ethephon in real-world settings, reducing the N application rates and increasing plant density to an optimal level is likely to improve maize NUE.

## Data Availability Statement

The original contributions presented in the study are included in the article/Supplementary Material, further inquiries can be directed to the corresponding author/s.

## Author Contributions

YZ, DY, and MZ designed the experiment. YZ, YW, CL, DY, and DR conducted the experiment and analyzed the data. YZ wrote the draft. YZ, ZL, and MZ revised the manuscript. All authors contributed to the article and approved the submitted version.

## Funding

This research was supported by the National Nature Science Foundation of China [Grant Number 31871546].

## Conflict of Interest

The authors declare that the research was conducted in the absence of any commercial or financial relationships that could be construed as a potential conflict of interest.

## Publisher's Note

All claims expressed in this article are solely those of the authors and do not necessarily represent those of their affiliated organizations, or those of the publisher, the editors and the reviewers. Any product that may be evaluated in this article, or claim that may be made by its manufacturer, is not guaranteed or endorsed by the publisher.
