# Supplementary material for: Ethephon Reduces Maize Nitrogen Uptake but Improves Nitrogen Utilization in Zea mays L
Source: Front Plant Sci. 2022 Jan 11;12:762736. doi: 10.3389/fpls.2021.762736 (PMC8786810; doi:10.3389/fpls.2021.762736)
Supplement: Supplementary file 1 [file Data_Sheet_1.PDF]

Supplementary Material

1 Supplementary Tables

Table S1. Summary of regression analysis for relationships evaluated in this research

Y vs. X	Treatment	R^2	n	Slopes	95%CI of slopes	Intercepts	95%CI of intercepts
GY vs. BM	CT	0.66	96	0.52 *	(0.43, 0.6)	0.77	(-0.84, 2.36)
	ET	0.69	96	0.61	(0.52, 0.7)	-0.59	(-2.22, 1.05)
GY vs. NUT	CT	0.58	96	0.031 *	(0.025, 0.036)	5.0	(3.9, 6.1)
	ET	0.64	96	0.038	(0.032, 0.045)	4.0	(3.0, 5.0)
GY vs. PreN	CT	0.56	96	0.050	(0.040, 0.060)	5.3	(4.1, 6.3)
	ET	0.61	96	0.051	(0.041, 0.06)	4.9	(3.9, 5.9)
GY vs. PostN	CT	0.44	96	0.059	(0.044, 0.074)	6.3	(5.2, 7.4)
	ET	0.33	96	0.068	(0.046, 0.090)	6.3	(5.0, 7.5)
GY vs. RemN	CT	0.35	96	0.082	(0.057, 0.107)	7.1	(6.0, 8.3)
	ET	0.58	96	0.084	(0.068, 0.100)	5.9	(5.1, 6.8)
RemN vs. PreN	CT	0.56	96	0.36 *	(0.29, 0.43)	3.3	(-4.5, 11.1)
	ET	0.82	96	0.54	(0.48, 0.59)	-5.6	(-11.5, 0.3)
RemN vs. NC_{silk}	CT-FD	0.75	48	5.5 *	(4.3, 6.7)	-47.9	(-66.0, -29.8)
	ET-FD	0.94	48	7.3	(6.6, 8.0)	-69.1	(-80.4, -57.7)
	CT-AD	0.81	48	7.4	(6.4, 8.4)	-43.3	(-56.2, -30.5)
	ET-AD	0.88	48	7.1	(5.8, 8.4)	-41.7	(-59.1, -24.3)
GNC vs. NUT	CT	0.96	96	0.58 *	(0.56, 0.61)	9.2	(4.1, 14.3)
	ET	0.96	96	0.65	(0.62, 0.68)	2.6	(-2.3, 7.5)
GNC vs. BM	CT	0.83	96	8.6	(7.7, 9.4)	-48.2	(-64.8, -31.7)
	ET	0.83	96	9.3	(8.4, 10.3)	-56.7	(-73.3, -40.0)
GNC vs. GY	CT	0.64	96	11.3	(9.4, 13.2)	-10.9	(-32.1, 10.3)
	ET	0.71	96	11.2	(9.6, 12.8)	-12.4	(-29.5, 4.7)
NUT vs. BM	CT	0.78	96	13.9	(12.2, 15.6)	-83.9	(-115.5, -52.4)
	ET	0.78	96	13.7	(12.0, 15.3)	-79.0	(-107.6, -50.3)
PreN vs. PreBM	CT	0.72	96	18.0	(15.5, 20.5)	-32.2	(-51.6, -12.9)
	ET	0.65	96	21.1	(17.6, 24.5)	-43.7	(-68.1, -19.4)
PostBM vs. PostN	CT	0.68	96	0.071 *	(0.060, 0.082)	6.0	(5.2, 6.8)
	ET	0.62	96	0.090	(0.074, 0.106)	5.4	(4.5, 6.3)
RemN vs. PreBM	CT	0.69	96	8.5 *	(7.2, 9.7)	-23.6	(-33.4, -13.8)
	ET	0.67	96	12.6	(10.6, 14.6)	-38.3	(-52.2, -24.3)

Note: GY represented grain yield; GNC, grain N content; NUT, total N uptake; PreN, pre-silking N uptake; PostN, post-silking N uptake; RemN, post-silking N remobilization; NC_{silk}, plant N concentration at silking stage; BM, total biomass achieved at harvesting stage per unit area; PreBM, pre-silking biomass per unit area; PostBM, biomass accumulation after silking per unit area; $PreNO_3$, NO_3 fluxes in xylem bleeding sap at V13 stage; $PostNO_3$, NO_3 fluxes in xylem bleeding sap at 30 DAS. Data of different N rates and plant density under ET and CT were jointly used for regression

analysis. The correlation evaluated in this research were all significant at *P*-value < 0.001. Asterisk means the slopes of regression lines of ET and CT had significant difference based on F-test ($P < 0.05$).

Table S2 Maize grain yield and N use efficiency indexes under advanced plant density with ethephon and under farmers' plant density with control at N sufficient condition.

Treatment	Grain yield ($t\ ha^{-1}$)	HI (%)	NHI (%)	NAE ($kg\ kg^{-1}$)	NRE (%)	PFPN ($kg\ kg^{-1}$)	NUpE ($kg\ kg^{-1}$)	NIE ($kg\ kg^{-1}$)
CT-FD	10.9 b	53.0 b	61.1 b	15.4 b	41.1 a	60.7 a	1.15 a	52.7 b
ET-AD	11.5 a	56.2 a	67.2 a	19.8 a	48.4 a	63.8 a	1.03 a	61.6 a

Note: CT-FD represented control under farmer's plant density (6.75 plant m^{-2}) and ET-AD represented ethephon-treated maize under advanced plant density (7.5 plant m^{-2}). HI, harvest index; NHI, N harvest index. NAE, N agronomic efficiency; NRE, N recovery efficiency; PFPN, partial factor productivity of N; NUpE, N uptake efficiency; NIE, N internal efficiency. Numbers are means of corresponding treatment under N150 and N225 jointly. In each trait, different letters indicated significant difference determined by Fisher's least significant difference (LSD, *a* = 0.05).

Table S3 Lodging rate of each treatment during the experimental period.

Treatments			Lodging rate (%)		
			2014	2015	2017
FD	CK	N0	0	0	0
		N75	0	0	0
		N150	2.1	0	1.1
		N225	1.7	1.5	1.3
	ET	N0	0.3	0	0
		N75	0	0	0
		N150	0	0.5	0.8
		N225	1.7	0.3	0.3
AD	CK	N0	0	0	0
		N75	0	0	0
		N150	1.7	1.3	1.3
		N225	1.5	1.7	1.9
	ET	N0	0	0	0
		N75	0	0	0
		N150	0	0	0.5
		N225	0.3	0.3	0
